# Exploratory household food consumption patterns and their associations with socioeconomic characteristics in Thailand: analysis of 19 rounds of the national household socio – economic survey from 2000 to 2021

**DOI:** 10.1186/s12937-026-01353-3

**Published:** 2026-07-07

**Authors:** Sumaira Zafar, Muhammad Umar, Warangkana Srichamnong, Rajesh Kumar Rai, Keith Lividini, Sabri Bromage

**Affiliations:** 1 Community Nutrition Unit, Institute of Nutrition, Mahidol University, 999 Phutthamonthon 4 Road, Salaya, Phutthamonthon, Nakhon Pathom 73170 Thailand; 2 Food Chemistry Unit, Institute of Nutrition, Mahidol University, 999 Phutthamonthon 4 Road, Salaya, Phutthamonthon, Nakhon Pathom 73170 Thailand; 3 Human Nutrition Unit, Institute of Nutrition, Mahidol University, 999 Phutthamonthon 4 Road, Salaya, Phutthamonthon, Nakhon Pathom 73170 Thailand; 4 Birbhum Population Project, Society for Health and Demographic Surveillance, Suri, West Bengal 731101 India; 5 Division of Global Health Management and Policy, School of Public Health, San Diego State University, San Diego, CA 92182 USA; 6 Department of Global Health and Population, Harvard T.H. Chan School of Public Health, 665 Huntington Avenue, Boston, MA 02115 USA; 7 Independent Consultant, 88 V St SW, Washington, DC 20024 USA; 8 Department of Nutrition, Harvard T. H. Chan School of Public Health, 665 Huntington Avenue, Boston, MA 02115 USA

**Keywords:** Household consumption and expenditure, Diet patterns, Dietary surveillance, Principal component analysis, Spatiotemporal analysis, Urbanization, Nutrition transition, Public health policy, Nutritional epidemiology, Thailand

## Abstract

**Background:**

Thailand has experienced major shifts in food consumption patterns due to rapid socioeconomic development, urbanization, and globalization, with implications for population nutrition. However, empirical evidence on long-term patterns from survey data is scarce.

**Methods:**

We analyzed nationally representative household expenditure data on 13 food groups from the Annual Household Socio-Economic Survey (HSES) for 19 waves from 2000-2021, with three waves (2011, 2015, 2019) also including granular data on 257 individual foods. HDDI was calculated at the household level as the count of distinct food groups consumed, based on expenditure data, and to evaluate the nutritional significance of the patterns, HDDI was also correlated with pattern adherence. To all 13 food groups and 257 individual foods, we applied principal component analysis to derive household “food consumption” and “food composition” patterns (FCPs and FCoPs, based on daily expenditures and proportions of total household expenditures, respectively). Patterns were grouped into five diets and mapped to explore spatiotemporal differences and trends. Generalized linear model (GLM) regression was used to identify associations between patterns and household demographics and socioeconomics.

**Results:**

Analysis of 13 major food groups from 2000 to 2021 identified four main diet categories for FCPs and FCoPs: Traditional Thai meals, mixed/diversified diets, convenience/prepared foods, and alcohol/lifestyle-related diets. In a detailed analysis of 257 individual foods, five major diets were recognized: four remained consistent from 2000 to 2021, and a fifth was a meat-dominant diet that grouped all patterns with high loadings for meats, specifically red meat. Traditional Thai/home-cooked patterns remained prevalent in rural households and in the Northern and Northeastern regions, though they are gradually declining. Mixed/diversified diets were more common in urban areas, especially in the Bangkok Metropolitan Region (BMR), but adherence decreased in other regions. Consumption of convenience and prepared foods increased over time as urbanization and changing lifestyles progressed. Alcohol/lifestyle-related diets showed lower adherence, whereas meat-dominant diets were more prevalent in the Northern and Northeastern regions. HDDI was positively correlated with traditional and mixed diets but only weakly or negatively associated with convenience-based dietary patterns.

**Conclusions:**

Results reveal persisting consumption of traditional foods in Thai rural and low-income households, while urbanites increasingly consume mixed and convenience diets. Growing reliance on prepared foods and generational and regional differences in alcohol consumption patterns have important implications for public health and policy.

**Supplementary Information:**

The online version contains supplementary material available at https://doi.org/10.1186/s12937-026-01353-3.

## Introduction

Since the 1970s, Thailand has undergone a dramatic socio–economic transformation toward a rapidly modernizing upper – middle – income economy. This transformation was fueled by a series of major economic development plans, significant foreign investment, and rapid urbanization [1–3], which led to improved education opportunities, diversification of household (HH) livelihoods, and demographic shifts toward older and smaller HHs [4, 5]. Broad socioeconomic and demographic shifts, coupled with ongoing globalization of the food market, have driven a significant transformation in food production systems from a predominantly agrarian model to a more diversified and market–oriented system [6, 7].

These shifts, in turn, have implications for population nutrition. Thailand has made dramatic progress in reducing poverty and hunger since the successful integration of community–based nutrition programs in the national development strategy in the 1980s [8], but food security and micronutrient deficiencies remain significant challenges in certain regions and population subgroups [9–11]. More recently, Thailand is facing a rapidly escalating burden of non–communicable disease (NCD), including cardiovascular disease, type II diabetes, obesity, and cancer, the burden of which is exacerbated by an aging population, proliferation of highly processed and nutrient–scarce foods, and increasingly sedentary lifestyles [12, 13]. Three out of every four deaths in Thailand are now caused by NCDs, and the majority of these NCD deaths are attributable to dietary and metabolic risks [14, 15].

Despite the magnitude of the nutrition transition in Thailand, the shifts and patterns in the most proximal risk factor for malnutrition (i.e., diet) have not been studied comprehensively. Nationally representative data on detailed dietary measurements collected via 24–hour recall methods are infrequent, have varied in coverage of different life stages, and are fragmented across rounds of different periodic and ad hoc survey systems [16, 17]. Such systems have more commonly relied on food frequency questionnaires, of which the local validity and comparability are challenging to firmly establish [18]. Health and nutrition surveys are also not generally geared towards understanding the multitude of socioeconomic factors associated with shifts in population diets, which can provide a lens into interventions to improve diets.

As in many countries, Thailand collects periodic data on HH consumption and expenditure, including data on food [19]. Although these data face numerous important limitations in nutrition research [20], they are nonetheless nationally representative, assessed frequently, and use consistent methodology over a long period of time, and provide detailed socioeconomic and demographic information on HHs and HH members with which to contextualize food consumption [21]. In this study, we examined long–term changes in HH food consumption using 19 rounds of the Thailand Household Socio – Economic Survey (HSES) data spanning 2000–2021. Drawing on methods from nutritional epidemiology, we analyze temporal and spatial changes in exploratory HH food consumption patterns and their associations with other household characteristics. We apply these insights to inform policies for shifting populations toward healthier diets.

## Methods

This study aims to identify the food consumption (FCP) and composition pattern (FCoP) of Thailand, how these patterns have changed over the last 22 years, and to discuss possible explanations for these shifts. Furthermore, this study has used quantitative methods to identify the changes in food patterns between 2000 and 2021.

### Dataset

The National Thailand Household Socio–Economic Survey (HSES), administered by the National Statistical Office (NSO) since the 1960s, spans the breadth of urban and rural Thailand and captures a comprehensive array of data related to HH living conditions, including income, expenditure, education, health, housing, employment, and access to public services. HSES is a multistage cluster survey employing robust sampling methodology and is designed to provide statistics representative at national, regional, and provincial levels. Historical HSES data for this study were obtained from the NSO for 19 rounds between 2000 and 2021. Within each year, we merged the following files: records of location (Rec 01), records for HH characteristics (Rec 02 and 03), and records of food groups (Rec 07 from 2000 to 2006 and Rec 14 for the rest of the years) using households as the unit of analysis. All years were then combined to create the working dataset (Supplementary File 1, for data collection and related information).

For every year, HSES contains weekly data on expenditures (in THB) of 13 food groups: “(i) Grains and Cereals”, “(ii) Meat and Poultry”, “(iii) Fish and Seafood”, “(iv) Milk, Cheese, and Eggs”, “(v) Oil and Fats”, “(vi) Fruits and Nuts”, “(vii) Vegetables”, “(viii) Sugar and Sweets”, “(ix) Spices and Condiments”, “(x) Prepared Meals Taken Home”, ”(xi) Non–Alcoholic Beverages at Home”, “(xii) Alcoholic Beverages (at Home and Away from Home)”, and “(xiii) Meals Eaten Away from Home".

More granular data were also obtained on HH expenditures on specific foods for three years: 2011, 2015 and 2019 (257 foods). These foods were condensed into 113 individual foods based on similar nutritional and culinary characteristics, to aid interpretability of results (food groups were provided in Supplementary Table S1).

For both the 13 broader food groups and the 257 granular ones, weekly expenditures were converted to daily expenditures and further used to calculate the proportion of each aggregated and disaggregated food group in relation to total household expenditures.

### Household Diet Diversity Index (HDDI)

Dietary diversity indicators such as the HDDI are widely used proxies for diet quality and micronutrient adequacy in population studies [22]. Household Diet Diversity Index (HDDI) was adapted by grouping food consumption expenditure into eight nutritionally relevant food categories: grains and cereals, meat and poultry, fish and seafood, milk, cheese and eggs, oils and fats, fruits and nuts, vegetables, and sugar and sweets. Each food group was assigned a value of “1” if the household reported any expenditure on that food group during the reference period and “0” otherwise. The HDDI was calculated as the sum of the binary indicators, resulting in a score ranging from 0 to 8, where higher values indicate diverse household diets. Households were further categorized into three diet diversity levels based on the HDDI: low (0–3), medium (4–5), and high (6–8). Similarly, HDDI was calculated for 257 individual food items from 2011, 2015 and 2019 data (Supplementary File 2, for classification of food items into major food groups as per FAO guidelines [22]).

To examine temporal changes in dietary diversity, a linear regression model was estimated with the mean yearly HDDI as the dependent variable and year as the independent variable. This analysis was used to assess whether household dietary diversity exhibited a significant increasing or decreasing trend over the study period.

### Food consumption patterns

All statistical analyses were conducted in R v.4.3.1 [23]. Principal component analysis (PCA) was applied to derive exploratory/posteriori household food consumption patterns [21]. Patterns were derived using (1) all available years of data from 2000 to 2021 for which 13 broader food groups were available (providing a long–term mass of data with which to extract patterns), (2) as well as the 257 food items under the broader food groups processed for 2011, 2015, and 2019 (allowing us to better interpret the long–term analysis with results from more granular data). The patterns identified are the patterns of household food procurement strategies, rather than precise patterns of individual nutritional intake. In contrast to cluster analysis, which identifies mutually exclusive groups of households associated with specific dietary patterns, we use PCA to identify underlying dietary patterns in the population for which continuous adherence can be measured over time, and to identify the important socioeconomic factors associated with that adherence.

In both cases, two sets of patterns were generated, based on daily expenditures and on expenditures as a proportion of household totals. Generating patterns based on daily expenditures allowed us to understand the influence of both the quantity and composition of expenditures (“food consumption patterns”), while patterns based on the proportion of total expenditures allowed us to isolate “food composition patterns” (roughly analogous to crude and energy–adjusted analyses in nutrition studies) [24]. Using expenditure shares allows the analysis to capture the relative importance of different food groups within the household food basket, thereby reflecting variations in dietary composition across regions and years. Shares of each food group as a proportion of daily total household food expenditures are also provided for the most recent year of observation (2021) in Supplementary Table S8.

Patterns were generated using the prcomp function in the stats package [23]. Patterns were retained according to quantitative criteria (variance explained by each rotated factor, and all factors, based on their associated eigenvalues and inspection of a scree plot) and qualitative criteria (interpretability of each factor with respect to its combination of high and low factor loadings, and the distribution of adherence to each pattern across population subgroups). Factor loadings were used to calculate patterns scores for each participant household and scaled from 0 to 100. To assess the nutritional relevance of the derived patterns, we examined Pearson correlation coefficients between household pattern adherence and the Household Dietary Diversity Index (HDDI).

To assess the robustness of the food consumption patterns generated from the aggregated data across all years and regions, additional analyses were conducted by restricting pattern generation to various subsets of years and examining each region individually (Supplementary Tables S2 and S3). Furthermore, the impact of using household expenditures per capita in the pattern generation process was investigated. However, this approach yielded minimal monetary values, resulting in lower explained variance and indicating that the use of per capita expenditures did not meaningfully contribute to the robustness of the identified patterns.

### Descriptive and associational analyses

The survey datasets include household-level sampling weights that already adjust for the unequal probability of household selection. In this study, an extra scaling step was applied to enhance the comparability of weights across survey years and to address differences in sample size between datasets. First, household weights were standardized within each survey year using z-scores, and then rescaled so that the relative importance of observations is comparable across datasets. The adjusted weight is calculated as:

1 $$\:New\:weight=\frac{Weight-\:\mu\:Weight}{\sigma\:Weight}$$

where µWeight and σWeight are the mean and standard deviation of the household weights, respectively.

Second, to account for variations in the number of surveyed households across years, the standardized weights were further adjusted based on the average number of households across all survey years. This adjustment considers differences in sample size and therefore reflects the relative precision of each dataset. The adjusted weight is computed as:

2 $$\:\text{}\:Adj\:weight=new\:weight\times\:\:\frac{Average\:HH}{Count\:of\:HH}$$

where average HH is the average number of households surveyed from 2000 to 2021, and count HH is the number of households surveyed in each respective year.

The adjusted weights were incorporated into a survey design object using the `svydesign()` function from the survey package. The design accounts for stratification by year, with each household’s weight contributing to population–level inferences. The final survey design is defined as:

3 $$\begin{aligned} &\:Svydesign\:(ids=\:\sim1,\:strata\\&=Year,\:weights=adj\:weight,\:data\\&=survey\:data,\:\:\mathrm{n}\mathrm{e}\mathrm{s}\mathrm{t}\:=\:\mathrm{T}\mathrm{R}\mathrm{U}\mathrm{E}) \end{aligned}$$

Using the weight–adjusted data, mean adherence (calculated by summing the expenditures of all food groups, weighted by their factor loadings) to household food consumption (FCPs) and composition patterns (FCoPs) was calculated for both aggregated (2000–2021) and disaggregated (2011 and 2019) food data. These adherence values were derived as both daily expenditures and proportions of household totals, stratified by year, geographic area, and selected household characteristics (e.g., region, urban/rural locality, income, household head gender, education, No of HH members, and economically active members, guided by local literature [18], Appendix I).

The spatiotemporal distribution of mean adherence to retained FCPs and FCoPs was visualized over time through mapping and animation. This approach provides a comprehensive understanding of how adherence to these patterns evolved across different regions and time periods.

4 $$\begin{aligned} &\:Model<-\:svyglm\:(FCP/FCoP\\&\:\sim\:Age+Gender+Education+members+\:EcoActiveMembers\\&\:+Current\:Income\:+\:Food\:Consump\:Expense\:+\:Year\\&\:+\:Province,\:\:design\:=\:design\_data) \end{aligned}$$

A survey-weighted generalized linear model (GLM) was designed to explore the factors influencing household food consumption and composition patterns. The model incorporates several predictors: the age, gender, and education of the household head, household size (number of members), the count of economically active members (EcoActiveMembers), total current household income, and monthly food consumption expenses per HH. Additionally, it includes the year of observation to account for temporal changes and the provincial location to capture spatial variations. By using the `svyglm` function, the model accounts for the survey design, yielding more robust estimates that reflect the complex sampling structure of the data. The outcomes of this analysis aim to elucidate the significant (with *p* value of < 0.05) determinants of HH food consumption patterns, providing insights into socioeconomic influences on dietary behaviors.

## Results

### Household Diet Diversity Index (HDDI)

**Fig. 1:**
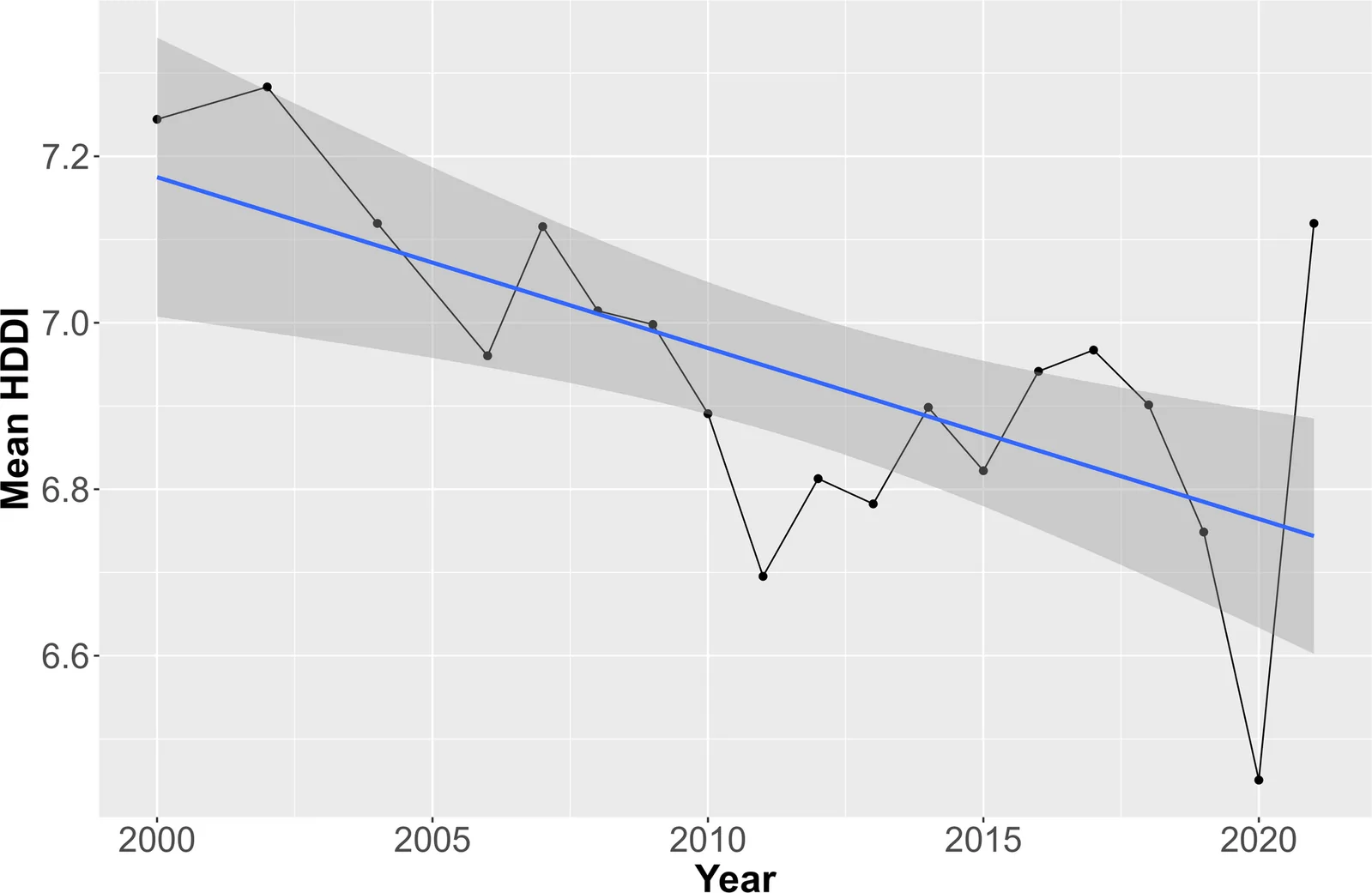
Temporal trend in Household Diet Diversity Index – HDDI (2000–2021)

**Fig. 2:**
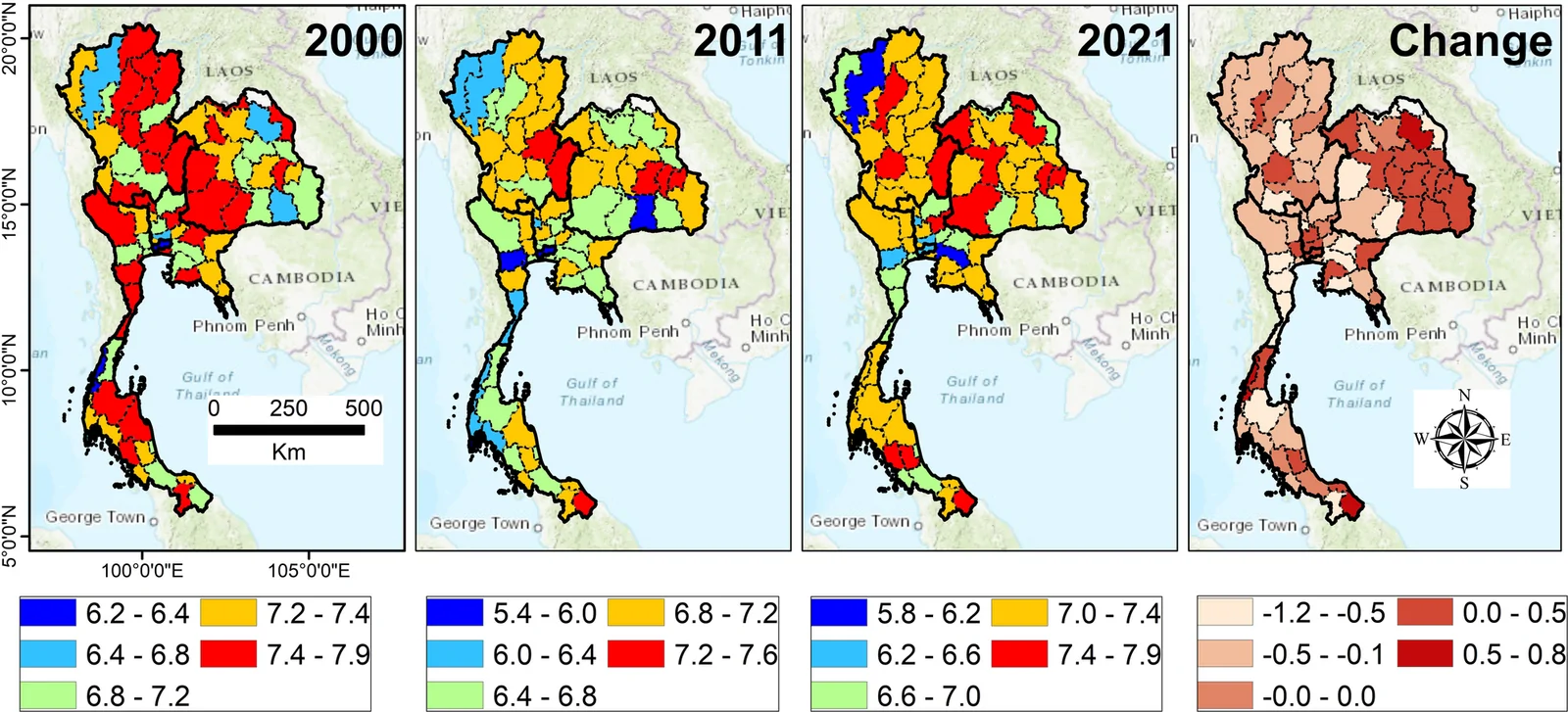
Spatial change in Household Diet Diversity Index – HDDI with 13 aggregated groups for 2000 and 2021, and 257 individual food items for 2011, 2015, 2019

The temporal trend presented in Fig. 1 shows a gradual downward trend in mean HDDI from approximately 7.2 in 2000 to below 6.8 in the late 2010s. Despite short-term fluctuations, the fitted regression line indicates a consistent decline in household dietary diversity over time. A sharp decrease is observed around 2020, followed by a partial rebound in 2021; however, the overall trend remains negative, suggesting a gradual deterioration in HDDI across Thailand over the past two decades. Fig. 2 shows the spatial distribution of mean Household Diet Diversity Index (HDDI) across Thailand in 2000, 2011, and 2021, together with the provincial and regional changes over time. In 2000, higher levels of dietary diversity were observed in several provinces in the central region and parts of the North, while some provinces in the Northeast and the southern region had relatively low values. By 2011 and 2021, the spatial pattern indicates an overall decline in HDDI across much of the country. The change map further confirms that declines in dietary diversity were widespread, while only a few provinces in the Northeastern region exhibited improvements during this period.

### Food consumption (FCP) and composition (FCoP) patterns

#### 2000–2021

According to the scree plot and the interpretability of the loading factors, three major food consumption patterns out of ten (10) principal components (PCs) were clearly identified among Thai HHs (Table 1). Identified patterns were grouped into four broader categories to interpret dietary behaviors among the Thai population: (1) traditional Thai meals, (2) mixed/diversified diets, (3) convenience/prepared foods, and (4) alcohol/lifestyle–related consumption. These categories capture the dominant diet choices in both Food Consumption Patterns (FCP) and Food Composition Patterns (FCoP), derived from the aggregated 2000–2021.

#### 2011, 2015 and 2019

PCA was performed for the detailed data of per HH per day using 257 items under 13 major groups. To reduce the complexity of the PCA, these items were regrouped into 113 items, and four distinct PCs were selected. (Supplementary Tables S4 and S5). Identified patterns were grouped into five broader categories to interpret dietary behaviors. The fifth category is (5) Meat-dominant diets and the rest is the same as explained above for 2000–2021.

#### Traditional Thai meals

##### 2000–2021

These patterns emphase home-cooked foods made from basic ingredients. Meals prepared at home (MPH-FCP) show high positive loadings (≥ 0.2) on grains and cereals, meat and poultry, fish and seafood, oils and fats, and vegetables, indicating a strong reliance on Traditional home-cooked meals. Similarly, the MPH-FCoP shows high positive loadings (> 0.2) on similar food groups including spices and condiments, with negative loadings for PMTH, beverages, and MEAH (Table 1).

**Table 1:** Factor loadings for retained household food consumption and composition patterns generated using food groups expenditure data (2000–2021)

Food Groups	Food Consumption Patterns FCP				Food Composition Patterns FCoP	
	Mixed pattern	Meals prepared at home (MPH)	Spirits	Meals prepared at home (MPH)	Spirits and meals away home (SMEAH)	Prepared Meals taken home (PMTH)
	PC1	PC2	PC3	PC1	PC2	PC3
Grains and Cereal	0.64	0.30	0.03	0.62	0.17	0.22
Meat and Poultry	0.68	0.29	0.06	0.66	0.14	0.10
Fish and Seafood	0.64	0.24	0.02	0.58	–0.02	0.13
Milk, Cheese and Eggs	0.57	–0.04	–0.15	0.20	–0.13	–0.53
Oils and Fats	0.66	0.20	0.02	0.57	–0.09	–0.19
Fruits and Nuts	0.58	–0.28	–0.16	0.04	–0.58	–0.10
Vegetables	0.53	0.32	0.05	0.71	0.05	0.16
Sugar and Sweets	0.50	–0.16	–0.22	0.10	–0.40	–0.52
Spices and Condiments	0.75	0.11	0.02	0.60	–0.05	–0.14
Prepared Meals Taken Home (PMTH)	0.28	–0.62	–0.28	–0.54	–0.41	0.54
Non–Alcoholic Beverage at Home	0.29	–0.47	–0.13	–0.22	–0.28	0.05
Alcoholic Beverage at Home	0.26	–0.24	0.60	–0.10	0.31	0.00
Alcoholic Beverage Away Home	0.17	– 0.31	0.71	–0.15	0.45	–0.09
Meals Eaten Away Home (MEAH)	0.40	–0.56	0.01	–0.56	0.37	–0.37
Proportion of Variance	27.85	11.1	7.583	22.8	8.932	8.243

##### 2011, 2015 and 2019

Four home-cooked meal patterns were identified, which correspond to traditional Thai foods, mainly focused on rice, fish, vegetables, and traditional condiments. The Traditional Thai Meals (TTM-FCP), showed high loadings on glutinous rice (0.14), pork (0.2), and fermented fish (0.22), which are major components of traditional Thai culinary practices. Similarly, the TTM-FCP includes a wide variety of food items, reflecting a balanced home-cooked meal structure typical of Thai cuisine (Supplementary Table S4). The TTM-FCoP also highlights traditional meals centered on ten spices and condiments among the twenty identified food items. It shows high loadings > 0.3 on rice, vegetable oils, onions, garlic, and white sugars. This pattern also includes prepared coffee, other prepared foods, and semi-prepared noodles. Additionally, the Vegetarian and Seafood Food (Vege-Seafood) shows strong loadings for cruciferous vegetables, dark green leafy vegetables, culinary herbs, tomatoes, legumes, gourds, green onions, and seafood items such as fish and shrimp (Supplementary Table S4).

#### Mixed/diversified diets

##### 2000–2021

This pattern represents dietary diversity with home cooked meals and convenience foods. The mixed pattern (FCP) is characterized by high positive loadings (> 0.5) for food groups such as grains and cereals, meat and poultry, fish and seafood, milk, cheese, eggs, oils and fats, fruits and nuts, vegetables, sugar and sweets, and spices and condiments. This indicates a focus on home–prepared food. It also has factor loadings > 0.2 for PMTH and MEAH, suggesting a diverse dietary pattern that combines home-cooked meals with occasional consumption of pre-prepared or eaten-out meals (Table 1).

##### 2011, 2015 and 2019

The Mixed pattern-FCoP from aggregated data is characterized by high positive loadings (> 0.1) on a wide variety of food items, including grains and cereals, meat and poultry, fish and seafood, milk, cheese, eggs, oils and fats, fruits and nuts, vegetables, sugar and sweets, and spices and condiments, indicating a focus on home-prepared food. The inclusion of PMTH, MEAH, and alcohols shows diversity in diet by combining traditional foods with modern consumption behaviors.

#### Convenience/prepared foods

##### 2000–2021

This category is associated with convenience foods, prepared meals, takeaway and eating outside the home. Prepared Meals Taken Home (PMTH-FCoP) shows a positive loading on grains and cereals (0.22), PMTH (0.54), and negative loadings on milk, cheese, eggs, and other food groups. This pattern suggests an increased dependence on convenience foods, possibly reflecting a busy lifestyle or altered food preferences (Table 1).

##### 2011, 2015 and 2019

The Traditional Condiment and Prepared Food (TCPF-FCoP) from disaggregated data includes noodles (0.34), green papaya salad (0.4), and prepared foods (0.42), as well as glutinous rice, fermented fish, and traditional condiments, indicating a preference for strongly flavored meals. The Seafood and Prepared Foods (SPF-FCP) from aggregated data reflects a diet combining seafood and prepared foods, including shrimp, processed seafood items, prepared foods, and seasoning pastes. Two additional patterns further highlight convenience-focused dietary choices. The Meals Eaten Away from Home and Prepared Food (MEAH-PF- FCoP) from aggregated data shows high loadings for prepared foods, beverages, and meals consumed outside the home, especially breakfast, lunch, and dinner purchased from food vendors or restaurants. Similarly, the Meals Eaten Away from Home (MEAH) pattern from FCoP aggregated data emphasizes frequent eating out, with strong loadings for meals eaten away from home (breakfast, lunch, and dinner), and related food items such as glutinous rice and beer.

#### Alcohol/lifestyle–related diets

##### 2000–2021

This category includes dietary patterns associated with alcohol and Spirits consumption and lifestyle-related food choices. Two patterns show dietary behaviors associated with alcohol consumption and social activities. Spirits and Meals Eaten Away Home (SMEAH-FCoP) shows high positive loadings (> 0.3) on alcoholic drinks consumed at or away from home and MEAH, with negative loadings on various other food groups. Similarly, the Spirits (FCP) pattern indicates a strong tendency toward eating out and drinking alcohol outside the home, emphasizing a social or convenience–based dining style. Furthermore, the Spirits pattern has high positive loadings (> 0.6) on alcoholic beverages consumed both at home and away, with low or negative loadings on other food groups. This pattern reflects a lifestyle characterized by heavy drinking (Table 1).

##### 2011, 2015 and 2019

The Prepared Food and Spirits (PF-Spirits-FCP) from disaggregated data shows positive loadings primarily for fast food items, PMTH, MEAH, and alcoholic beverages, reflecting a lifestyle that includes alcohol consumption along with convenience foods. The Traditional Thai Meals with High Sugar (TTMHS-FCoP) from disaggregated data represents a home-prepared meal structure similar to traditional Thai diets but with higher loadings for sugar intake, especially white and palm sugars.

#### Meat-dominant diets

##### 2011, 2015, and 2019

Includes five dietary patterns centered on individual foods, mainly meat combined with vegetables or seafood. The Traditional Thai Meat Meals (TTMM-FCP) pattern from FCP disaggregated data includes high loadings for pork skin, beef skin, fresh chicken, fermented fish, glutinous rice, and various vegetables and spices, reflecting a meat-centered variation of traditional Thai cuisine. Three additional patterns from FCoP disaggregated data further illustrate meat-based eating habits. The Meat and Vegetable Meals (MVM) pattern shows high loadings for pork, chicken, and numerous vegetables, including Chinese broccoli, string beans, chili peppers, and spring onions. The Meat and Seafood Meals (MSM) pattern emphasizes a combinations of seafood with pork and cooked chicken, along with glutinous rice. Lastly, the Meat and Prepared Food (MPF) pattern highlights diets centered on pork and chicken, with ready-to-eat foods such as prepared noodles and green papaya salad, as well as carbonated drinks.

### Correlation of HDDI with patterns (2000–2021)

The Mixed and MPH patterns were positively correlated with HDDI, with the strongest association observed for the MPH pattern derived from FCoP (*r* = 0.55), followed by MPH from FCP (*r* = 0.44) and the Mixed pattern (*r* = 0.22). The Spirits pattern showed a weaker positive correlation (*r* = 0.15). In contrast, SMEAH (*r* = 0.03) and PMTH (*r* = − 0.19) showed little or negative association with dietary diversity (Table 2). This may partly reflect the lack of detailed ingredient information for meals eaten away from home and prepared meals taken home in the dataset, which could lead to their lower contribution to the HDDI calculation (Scatter plots in Supplementary File 3).

**Table 2:** Correlation of FCP and FCoP patterns adherence with HDDI

Category	Pattern type	Year	Patterns	*r*
Meals prepared at home/ Traditional Thai meals	FCP	2000–2021	MPH (PC2)	0.44
	FCoP	2000–2021	MPH (PC1)	0.55
	FCP (Aggregated data)	2011–2015,2019	TTM (PC2)	0.02
	FCP (Disaggregated data)	2011–2015,2019	TTM (PC3)	-0.02
	FCoP (Aggregated data)	2011–2015,2019	TTM (PC2)	-0.19
	FCP (Aggregated data)	2011–2015,2019	Vege-Seafood (PC3)	0.08
Mixed/diversified diets	FCoP (Aggregated data)	2011–2015,2019	Mixed pattern (PC1)	0.65
	FCP	2000–2021	Mixed pattern (PC1)	0.22
Convenience / prepared foods	FCoP	2000–2021	PMTH (PC3)	-0.19
	FCoP (Disaggregated data)	2011–2015,2019	TCPF (PC3)	-0.08
	FCP (Aggregated data)	2011–2015,2019	SPF (PC4)	0.11
	FCoP (Aggregated data)	2011–2015,2019	MEAH-PF (PC1)	0.14
	FCoP (Aggregated data)	2011–2015,2019	MEAH (PC4)	-0.44
Alcohol / lifestyle related	FCP	2000–2021	Spirits (PC3)	0.15
	FCoP	2000–2021	SMEAH (PC2)	0.03
	FCP (Disaggregated data)	2011–2015,2019	PF-Spirits (PC1)	-0.15
	FCoP (Disaggregated data)	2011–2015,2019	TTMHS (PC1)	0.15
Meat-dominant diets	FCP (Disaggregated data)	2011–2015,2019	TTMM (PC2)	0.1
	FCoP (Disaggregated data)	2011–2015,2019	MVM (PC3)	-0.35
	FCP (Disaggregated data)	2011–2015,2019	MVM (PC4)	-0.31
	FCoP (Disaggregated data)	2011–2015,2019	MSM (PC4)	-0.36
	FCoP (Disaggregated data)	2011–2015,2019	MPF (PC2)	0.1

### Spatiotemporal distribution of patterns (2000–2021 and 2011, 2015, 2019)

#### Meals prepared at home/ traditional Thai meals

##### 2000–2021

**Fig. 3:**
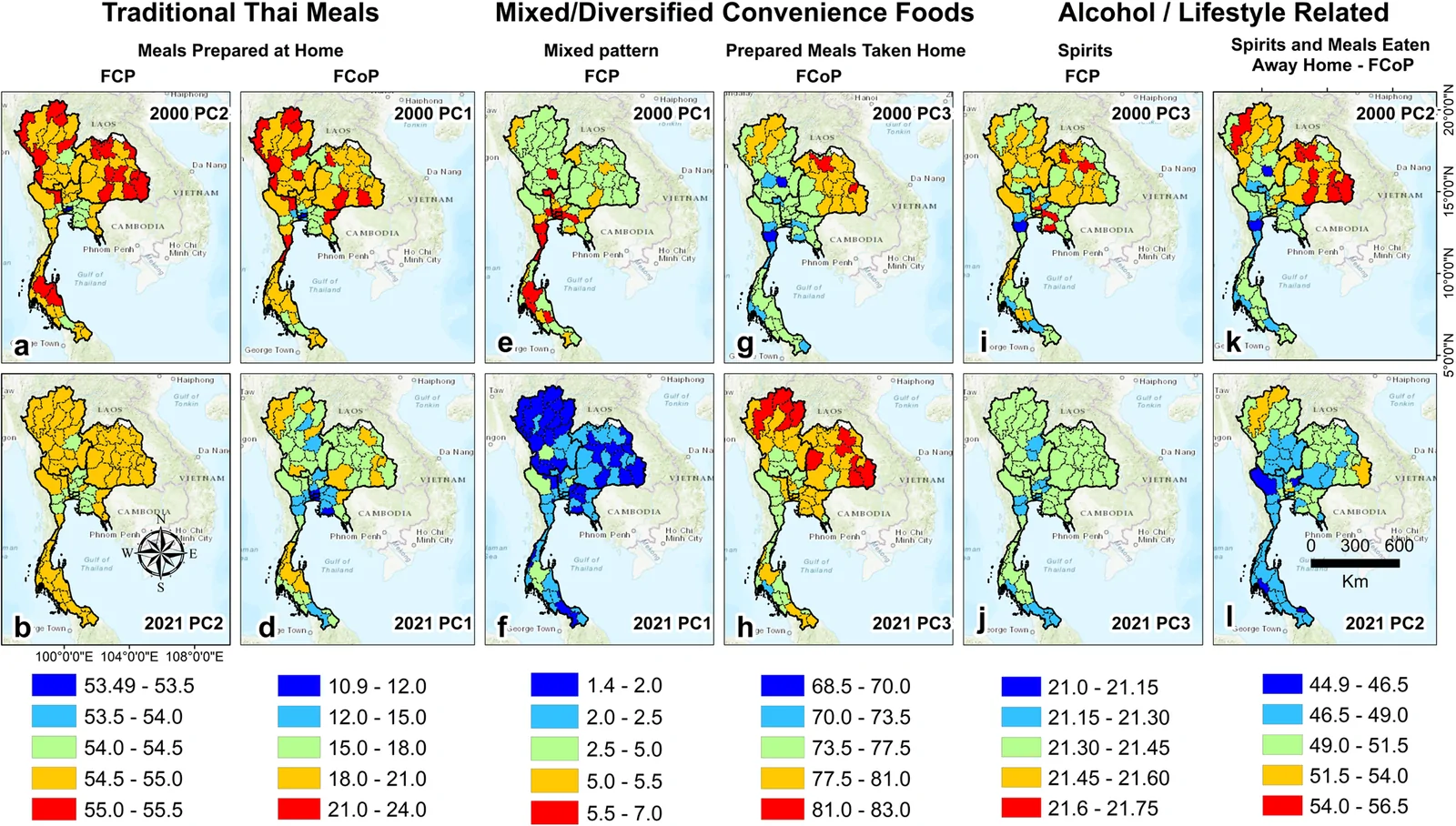
Mean adherence to food consumption (FCP) and food composition patterns (FCoP) under four categories by province in 2000 and 2021 Panels: (1)Traditional Thai Meals: Meals Prepared at Home FCPs (a, b) and Meals Prepared at Home FCoP (c, d); (2) Mixed/Diversified: Mixed Pattern FCP (e, f); (3) Convenience Foods: Prepared Meals Taken Home FCoP (g, h); and (4) Alcohol/Lifestyle-Related patterns: Spirits FCP (i, j) and Spirits and Meals Eaten Away from Home FCoP (k, l). For each pattern, the upper panel shows 2000 and the lower panel shows 2021 data.

Adherence to the MPH-FCP was high across most provinces, with comparatively lower levels in the BMR and neighboring provinces (Fig. 3a). The MPH pattern (Fig. 3b) adherence in 2021 increased in BMR and remained within the same range in adjacent provinces, but declined across Thailand.

Similar but more regionally differentiated distribution was observed for MPH-FCoP. In 2000 low adherence was observed to the MPH pattern in the BMR and adjacent provinces but high adherence in Northern, Northeastern, and Southern regions (Fig. 3c). By 2021, the adherence to the MPH pattern has generally decreased in most provinces, however the BMR shows an expansion of moderate adherence into adjacent provinces.

##### 2011, 2015, and 2019

**Fig. 4:**
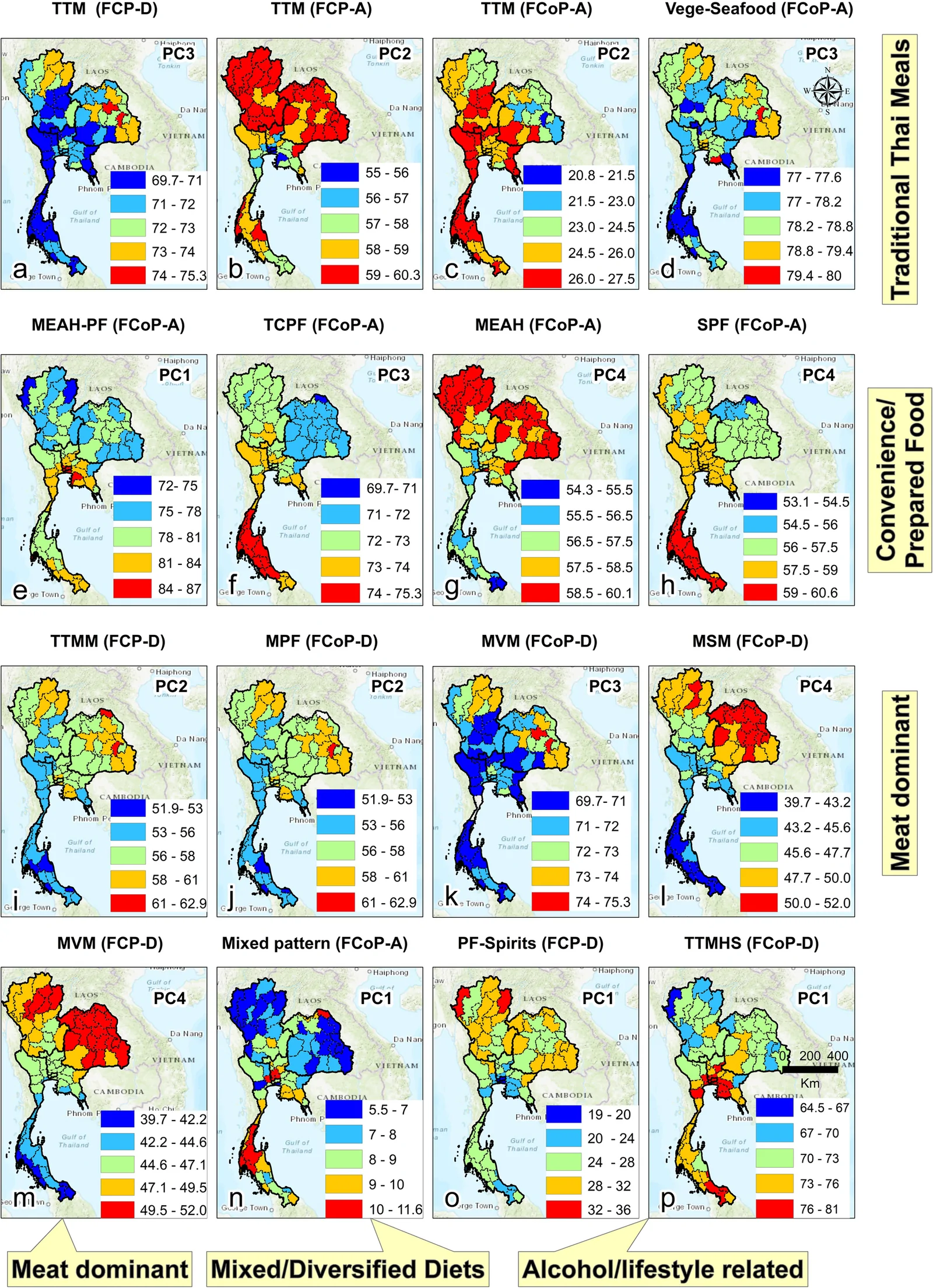
Mean adherence food consumption and composition patterns from disaggregated and aggregated data by province in 2011, 2015, and 2019 Panels: (1) Traditional Thai Meal patterns (a–d): TTM (FCP-D), TTM (FCP-A), TTM (FCoP-A), and Vege-Seafood (FCoP-A); (2) Convenience/Prepared Food patterns (e–h): MEAH-PF (FCoP-A), TCPF (FCoP-A), MEAH (FCoP-A), and SPF (FCoP-A); (3) Meat-Dominant patterns (i–m): TTMM (FCP-D), MPF (FCoP-D), MVM (FCoP-D), MSM (FCoP-D), and MVM (FCP-D); (4) Mixed/Diversified (n): Mixed Pattern (FCoP-A); (5) Alcohol/Lifestyle-Related patterns (o, p): PF-Spirits (FCP-D), and TTMHS (FCoP-D). A and D denote patterns derived from aggregated and disaggregated data from 2011, 2015, and 2019, respectively.

Four patterns were identified (Table 2): three were TTM (basically home-cooked meals) and Vegetables and Seafood Meals (Vege-Seafood). TTM remained widely prominent, especially in the Northern and Northeastern regions, while Vege-Seafood was more concentrated in the BMR and adjacent provinces (Fig. 4a, b, c, d), highlighting a distinct urban dietary preference.

#### Mixed/diversified diets

##### 2000–2021

In 2000, the mixed pattern showed moderate adherence in provinces of Northern and Northeastern regions, while high adherence in Bangkok Metropolitan Region (BMR), as well as in parts of the Western, and Southern regions (Fig. 3e). By 2021, adherence to this pattern declined, with most provinces exhibiting low adherence (Fig. 3f), indicating nationwide reduction in this pattern over time.

##### 2011, 2015, and 2019

during these years, the mixed pattern was found to be prominent in the neighboring provinces of the BMR and in the Southern region (Fig. 4p). This shows a more localized and regionally concentrated prevalence compared to its broader spatial distribution in 2000.

#### Convenience / prepared foods

##### 2000–2021

Adherence to the PMTH pattern in 2000, was highest in the Northern and Northeastern provinces and generally showed medium adherence across the country, with only few provinces exhibiting low adherence (Fig. 3g). By 2021, adherence to this pattern showed a further increased, particularly in the Eastern, Northern, and Northeastern provinces (Fig. 3h).

##### 2011, 2015, and 2019

in these years, four related patterns (MEAH-PF, TCPF, MEAH, and SPF) are identified, which remain concentrated in the BMR, as well as in the Eastern, Central, and Southern regions, reflecting urban lifestyles and tourism driven behaviors. Among these patterns, only MEAH had high adherence in the Northern and Northeastern regions, indicating a spread beyond urban centers (Fig. 4e, f, g, h).

#### Alcohol/ lifestyle-related diets

##### 2000–2021

The Spirits-FCP also exhibited moderate to high adherence in provinces of adjacent to BMR and in the Northern and Northeastern regions, while lower levels were observed in the Central and Southern regions (Fig. 3i). By 2021, adherence to this pattern remained unchanged or declined over the entire country (Fig. 3j), indicating an overall reduction across Thailand.

Similarly, SMEAH pattern showed low to moderate adherence in 2000, except in the Northern and Northeastern regions (Fig. 3k). By 2021, adherence further declined nationwide, suggesting a continued decrease in eating out dietary behavior over time. Overall this pattern showed a downward trend, despite some persistent regional differences (Fig. 3l).

##### 2011,2015, and 2019

Two patterns were included in this category: TTMHS presented highest adherence in the BMR, Eastern, and Southern regions, with moderate adherence across most provinces nationwide (Fig. 4i). PF-Spirits was the highest adherence in Northern and Northeastern regions (Fig. 4n, and 4o), indicating a distinct regional distribution compared to urban centered patterns.

#### Meat-dominant diets

##### 2011,2015, and 2019

Five dietary patterns were identified in this category, including TTMM, MVM (FCP), MVM (FCoP), MPF, and MSM. These patterns showed consistent higher adherence in the Northern and Northeastern regions, and the lower in BMR and other parts of the country (Fig. 4i, j, k, l, m).

### Transformation path map of Thailand dietary patterns

**Fig. 5:**
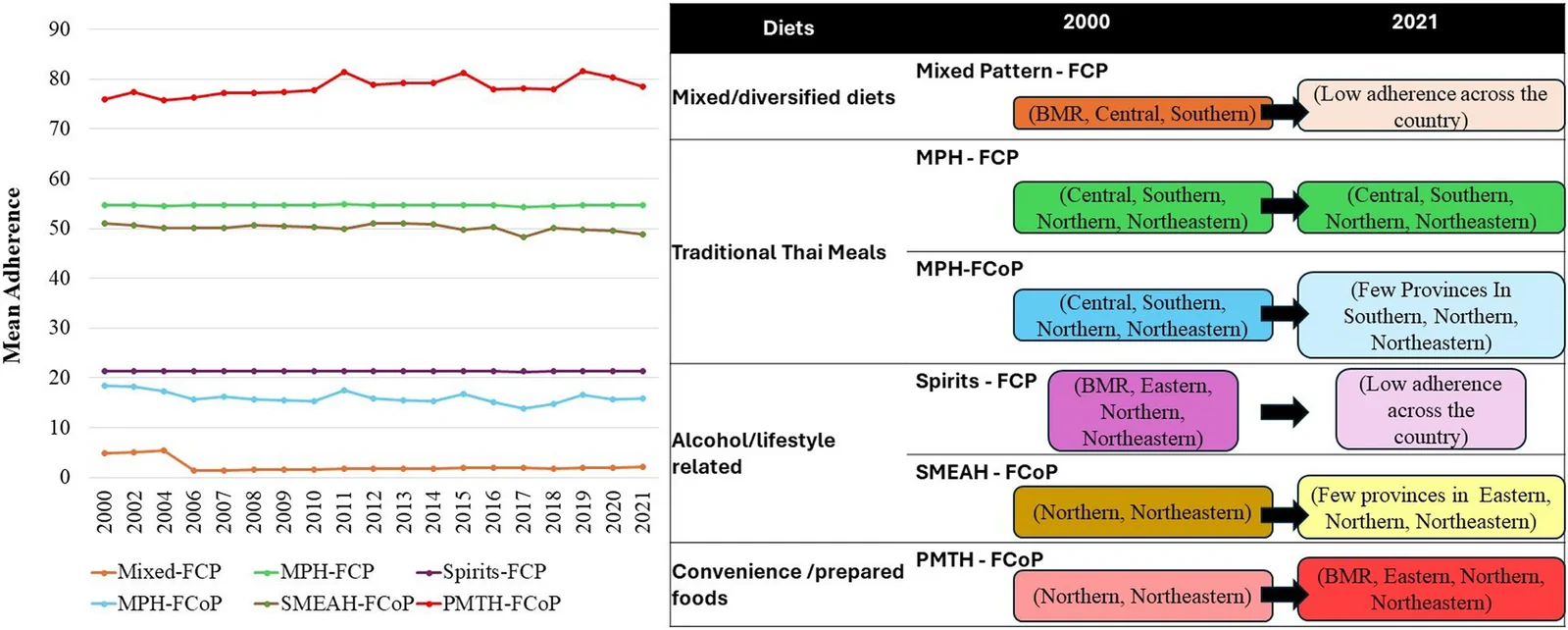
Transformation path map of dietary patterns in Thailand (2000–2021)”

A gradual dietary transition in Thailand was observed from long-term temporal trends in household adherence to dietary patterns (Fig. 5). Temporal trends in household adherence to dietary patterns reveal a gradual decline in traditional home-cooked meals and a rise in convenience foods. Adherence to meals prepared at home (MPH-FCoP) has increased over time, while mixed-FCP and MPH-FCP dietary patterns remained stable.

**Fig. 6:**
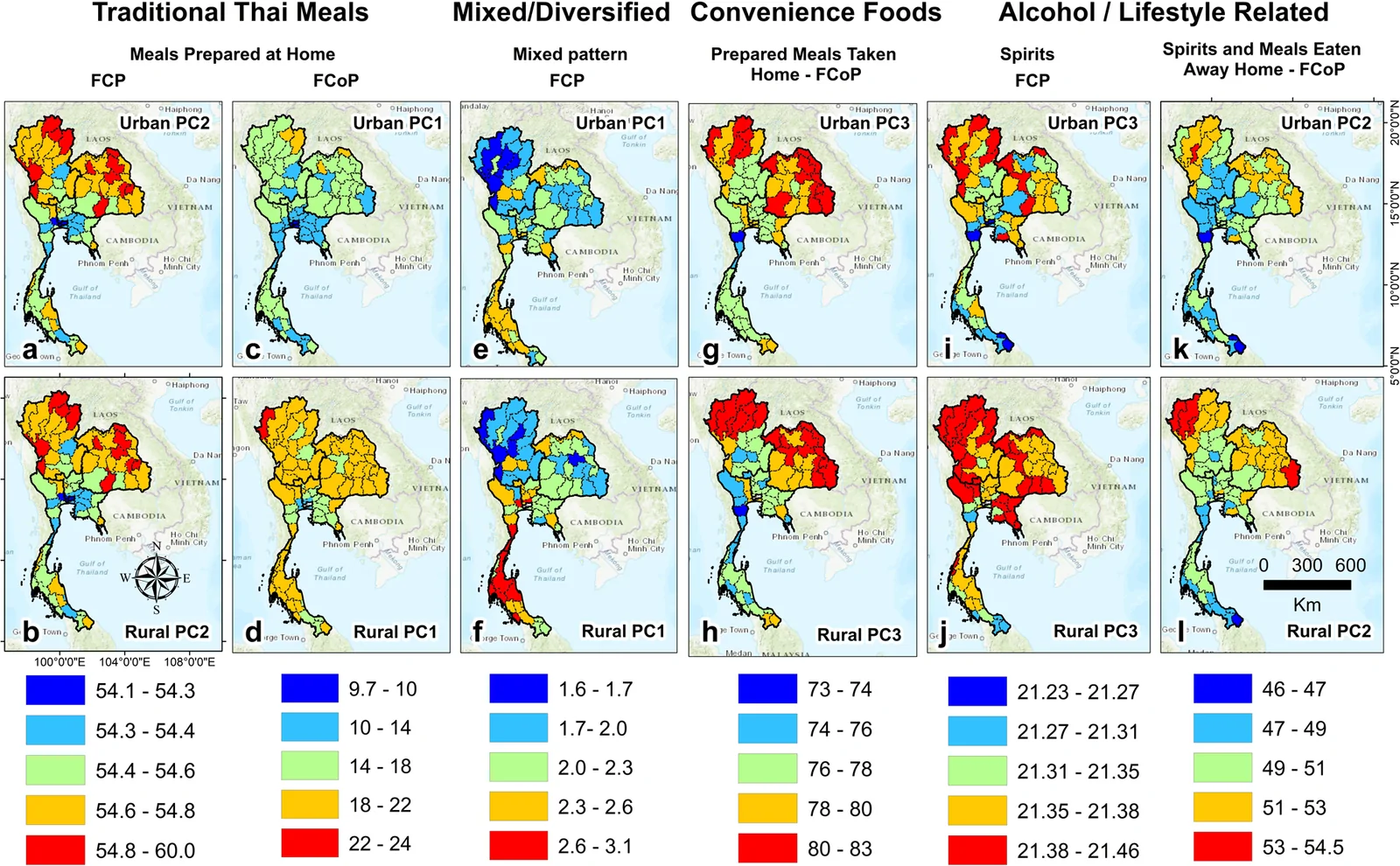
Urban–rural difference in mean adherence to household food consumption (FCP) and composition (FCoP) patterns under four categories by province for 2000 – 2021Panels in the upper row represent urban households, whereas panels in the lower row represent rural households. Panels: (1) Traditional Thai Meals pattern: Meals Prepared at Home FCP (a, b); Meals Prepared at Home FCoP (c, d); (2) Mixed Pattern FCP (e, f); (3) Convenience/Prepared Food patterns: Prepared Meals Taken Home FCoP (g, h); (4) Alcohol/Lifestyle-Related patterns: Spirits FCP (i, j) and Meals Eaten Away from Home FCoP (k, l).

Geographical shifts in pattern were also observed. Adherence to the mixed pattern decreased to its lowest level across the country in 2021 (lighter colors) compared to 2000, while it remained similar in the same regions for MPH-FCP (as shown in Fig. 6). However, MPH-FCoP showed a contraction in geographic coverage, with fewer provinces demonstrating adherence in 2021 than in 2000.

Alcohol-related dietary patterns SMEAH and Spirits also show reduced prevalence spatially (Fig. 3i, j, k, l) and temporally (Fig. 5). In contrast, the convenience/prepared foods dietary pattern increased in mean adherence across regions (Fig. 3g, h) and expanded geographically into additional regions (Fig. 5).

The transition pathway is characterized by the persistence of home-cooked meals alongside a broader shift away from traditional and alcohol-related dietary patterns toward increased adoption of a convenience lifestyle.

### Rural–urban spatiotemporal differences (FCP and FCoP, 2000–2021)

#### Meals prepared at home/traditional Thai meals

The MPH-FCP pattern showed the lowest adherence among urban households in BMR, moderate adherence in adjacent provinces, and the highest in the Northern and Northeastern regions (Fig. 6a). A similar spatial pattern was observed among rural households, with adherence in the Southern region (Fig. 6b). For MPH-FCoP, urban households showed low adherence in BMR and its neighboring provinces (Fig. 6c), reflecting metropolitan characteristics, while adherence was higher among rural households in all other regions except in provinces adjacent to BMR, indicating a tendency to cook meals at home (Fig. 6d).

#### Mixed/diversified diets

The Mixed pattern-FCPs showed the highest adherence among urban households (Fig. 6e) in the BMR and Southern regions (2.3–3.1%) and showed a similar pattern in rural households, but with higher adherence (Fig. 6f).

#### Convenience / prepared foods

The PMTH-FCP presented highest values in the BMR, Northern, and Northeastern regions (Fig. 6g). The cluster for rural households in the Northern and Northeastern regions shows higher adherence to PMTH than urban households (Fig. 6h). Indicating growing prevalence of prepared/convenience food beyond urban centers.

#### Alcohol/lifestyle-related

Spirit-FCP pattern showed the highest adherence, clustered in the Eastern, Northern, and Northeastern regions, while low values were observed in BMR and its adjacent provinces (Fig. 6i). However, for rural households, the pattern showed higher values across Thailand, except in a few provinces (Fig. 6j). Similarly, SMEAH-FCoPs also showed clustering of high adherence in the North and Northeastern regions and low and medium adherence in the rest of the regions for urban households (Fig. 6k). Among rural households, this pattern extended into a larger number of provinces with high adherence (Fig. 6l), showing broader rural distribution.

### Association analysis

The Association analysis reveals associations between household’s food consumption/composition patterns and several demographic and economic factors, and their relationship with food consumption patterns (FCPs) and food composition patterns (FCoPs). Model goodness-of-fit statistics (Pseudo R², AIC, and sample size) are reported by dietary pattern in Supplementary Tables S6 and S7. Model fit varied across dietary patterns, with mixed and convenience-related patterns showing higher explanatory power than traditional and alcohol-related patterns. In addition, p-values for demographic and economic factors are reported for each model. Interaction terms were tested but not retained as they were not statistically significant and did not improve model fit (ΔAIC).

The association analysis of pattern adherence with household demographics, 2000–2021, indicated three dietary transitions. First, household demographics are a major driver of dietary choices. Larger households with more economically active members are associated with high adherence to mixed and convenience diets, which correspond with higher HDDI levels, indicating diet diversity. On the contrary, a negative or weak positive association was found with Traditional Thai meals and alcohol/lifestyle. Second, a household’s socioeconomic status shapes diets in nuanced ways. Households with higher income are positively associated with mixed/diversified diets but negatively associated with traditional Thai meals and alcohol/lifestyle-related dietary patterns. Monthly food expenditure consistently drives convenience/prepared foods and alcohol/lifestyle-related dietary patterns, which also correspond to HDDI. This suggests that high food spending translates into diverse dietary choices, but again not necessarily into a balanced diet. Third, education and gender showed consistent negative associations across most patterns, suggesting limited differentiation in dietary behavior along these dimensions. Age shows a gradual shift away from alcohol/lifestyle-related diets and toward more traditional meals (Supplementary Table S6).

The findings from both aggregated and disaggregated (FCPs and FCoPs) models (2011–2019) complement results from long-term patterns (2000–2021), which confirm the role of economic status and household structure in shaping dietary transitions in Thailand. Across both aggregated and disaggregated FCPs and FCoPs, mixed, meat-dominant, and convenience/prepared foods (e.g., Mixed Pattern, Meat and PF, Seafood and Prepared Food) consistently show high R² up to 0.66, and are positively associated with economically active members and food expenditure, indicating that household members’ participation and purchasing power drive shifts toward convenience-based diverse diets. In contrast, traditional Thai meals exhibit weaker or negative associations with income and food expenditure, reflecting a declining priority. Education and gender generally maintain negative or insignificant associations, consistent with the findings from 2000 to 2021. These dynamics align with HDDI trends, in which patterns are linked to higher food spending, and diversification corresponds to higher dietary diversity, while traditional, less diversified patterns remain weakly associated (Supplementary Table S7).

## Discussion

Diets can be aggregated in different ways, from the level of individual nutrients and other bioactive compounds; foods and ingredients, meals, dishes, and food groups, to broad dietary patterns and metrics [25]. Arguments for examining dietary patterns are that patterns can capture synergistic interactions between foods and nutrients not otherwise observable using more reductionist approaches, and because patterns essentially describe lifestyles (e.g., adherence to a Western, traditional, or a healthy diet pattern, etc.) [26]. Results from pattern analysis may be more useful when translating information to the public. Prior studies conducted in Thai population [12, 27–31] derived exploratory diet patterns by applying principal component analysis to food or food group–frequency data (in some cases, based on a rather limited number of food groups) and the associations explored of the derived patterns with NCD-related outcomes across sociodemographic characteristics. To our knowledge, no prior studies for Thailand have developed the food consumption/composition patterns from NSO data (2000–2021). Because these patterns are derived from expenditures and expenditure shares rather than dietary intake, they should be interpreted as household food purchasing behaviors that may influence diet quality, rather than direct measures of nutritional intake.

The study finds a consistent dietary transition in Thailand over the past two decades, characterized by a gradual decline in household diet diversity alongside a shift in dominant food consumption patterns. This study is the first to investigate HDDI in Thailand from NSO data, revealing that diet diversity is declining in Thailand, except in Northeastern region. The decrease in HDDI was modest but consistent, indicating a shift in diets, which may be linked to increased consumption of energy-dense (rice, fats and oils) food rather than nutrient rich (vegetables, fruits, cereals, nuts, and eggs) due to changing lifestyles with urbanization and market dynamics; this is supported in part by a modest inverse correlation between PMTH and SMEAH vs. the HDDI. Although Bennett’s Law predicts a decline in the share of starchy staples as incomes rise, this relationship may be weaker in settings where staple foods remain culturally central. In Thailand, rice continues to play a dominant role across income groups, and rising incomes tend to increase dietary diversity through greater consumption of higher-value foods such as animal products and prepared meals rather than substantially reducing staple consumption [32].

The HDDI is a measure of how accessible diverse foods are, but this trend in Thailand raises concerns about long-term nutritional adequacy. A study from Thailand reported that older adults with high educational level, wealth index, and living in an urban area had higher diet diversity scores [33]. The drop in HDDI in 2020 was likely associated with COVID-19 disruptions, driven by reduced incomes, access constraints, and supply chain disruptions. A partial recovery was observed in 2021, but HDDI levels did not return to pre-COVID-19 levels, as the pandemic caused intensified pre-existing trends. Studies from other countries reported food insecurity among COVID-19 patients in Saudi Arabia [34], and sample population in Malaysia and Vietnam [35–37]. Correlation of HDDI with patterns, showed that dietary diversity is primarily linked with Traditional Thai meals and mixed/diversified diets, suggesting that these diets incorporate a range of food groups through home cooking which contribute diversity in diet and balanced food composition. In contrast, weak or negative relationships were found with PMTH and SMEAH patterns, showing that reliance on convenience/prepared foods contributes less to dietary diversity; this is likely due to limited ingredient variety and data constraints in capturing their full composition. A weak positive association with Alcohol/lifestyle related pattern further suggests that alcohol-related consumption does not meaningfully enhance dietary diversity.

Patterns from NSO data were grouped into five categories, (i) traditional Thai meals, (ii) mixed/diversified diets, (iii) Convenience/prepared foods, (iv) alcohol/lifestyle–related diets and (v) meat dominant diets. Traditional Thai meals grouped all the patterns identified as meals prepared at home (MPH) or Traditional Thai meals which focuses on individual food groups/ingredients that are likely to be used in home cooking. MPH remained persistent in most provinces of Northern and Northeastern Thailand and was still the most prevalent across Thailand (Fig. 3). The adherence remained consistently low in BMR (Fig. 4) for MPH-FCoP. As metropolitan trendsetter, BMR likely influences neighboring areas. Lifestyle changes, long hours and less time for cooking may further explain these shifts. The MPH-FCP adherence remained almost unchanged with a little decrease of 0.5% in most of the provinces of Northern and Northeastern Thailand and still, was the most prevalent across Thailand (Fig. 3). The finding that the MPH (FCP) pattern was a growing preference, specifically in BMR (increment of 1% between 2000 and 2021) aligns with increasing health consciousness and awareness of the benefits of home–cooked meals (during and after COVID 19), which were perceived to be healthier and more nutritious than processed or prepared options which sometimes may not be prepared hygienically [28, 38], raising the concern of food safety. On the contrary, MPH (FCoPs) adherence decreased across Thailand, particularly in the North and Northeast (by up to 3%), while adherence in BMR remained consistently low (Fig. 4).

The apparent increase in adherence to MPH patterns in BMR, despite a decline in traditional diets, can be explained by the distinction between absolute and relative food expenditures. The MPH-FCP is derived from absolute spending on food groups (in Thai Baht). MPH-FCoP is calculated as the proportional share of total food expenditure for each food group. The increase in adherence to MPH-FCP in BMR suggests that households are spending more in absolute terms on home-cooking ingredients. This is likely driven by higher incomes, greater health awareness, and behavioral shifts during and after the COVID-19 pandemic. However, the decline in adherence to MPH-FCoP indicates that the percent share of total food expenditure allocated to MPH has decreased. Expenditures on convenience/prepared foods and on dining out have sharply increased. This indicates a diversification of food consumption rather than a return to traditional diets. Urban households now combine home cooking with modern food choices. This contradiction highlights the transition pathway toward mixed and diversified dietary behaviors rather than a simple increase in adherence.

MPH and TTM patterns remain prevalent in rural areas among lower-income households, while urban and wealthier households show more diverse and mixed consumption preferences. These patterns were predominantly clustered in Northern and Northeastern regions, valued for their rich flavors and nutritional benefits. MPH and TTM exhibit significant positive associations with age, household size, and the number of economically active members, suggesting that larger households and those with more working members tend to adhere more closely to this diet. Isan youth showed a strong preference for local foods over fast food, emphasizing traditional dietary practices [39]. Earlier research also reported that older Thai adults have better health practices than younger adults [12, 30, 40]. Similarly, another study analyzed the food consumption behaviors and associated factors in the elderly in the northeastern region and reported that the elderly with higher education tend to have better knowledge of a healthy diet, therefore leading to better food consumption behaviors [33, 41, 42]. This trend aligns with the results of this study, which show a positive association between household head age and MPH/TTM (Supplementary Tables S6 and S7), indicating that they stick to the dietary practices they grew up with. Generational food preferences are shifting with older generations concerned about younger ones favoring non–nutrient–dense foods (e.g., butter, chocolate, chips, candy, and soda) due to the rise of Western–style foods and brands [43–45]. Younger generations in indigenous communities of West Sumatra, Indonesia, often avoid certain traditional wild plants due to their taste [43]. In Northeastern Thailand [41] and Jakarta Indonesia [46], living in extended or multigenerational households was linked to better food consumption behaviors compared to living alone. However, several articles highlight traditional diets as the most preferred food choice [47–49].

The Mixed/diversified diets include a wide array of food groups, suggesting a mix of homemade and eaten-out or takeout meals. The Mixed patterns diversity highlights households’ preference for varied diets, shaped by urbanization, evolving work conditions, and tourism [50]. Its dominance in urban higher–income households reflects how modernization influences food choices. Both Mixed patterns from aggregated data (2000–2021) and from detailed data (2011, 2015, and 2019) are dominant in the BMR and are associated with large families of higher incomes (*p* < 0.01) and higher food consumption expenses.

A growing reliance on convenience/prepared foods, including PMTH and MEAH, reflects contemporary lifestyle changes. The daily expenses on MEAH and PMTH account for almost 30% of food consumption expenses for Thai household (% daily share of expenditure on each food group per household, Appendix II). Prepared food consumption and dining out have become an integral part of Thai culture, particularly in urban areas and among younger generations. Six distinct patterns of prepared foods and eating out have emerged, including FCoPs (PMTH, MEAH-PF, MPF, TCPF, SPF, and MEAH), underscoring a growing reliance on convenience foods driven by time constraints and practicality [51]. Food choices may be healthy, but many prepared meals are high in unhealthy fats, sodium, and preservatives [52]. High income and a female household head were found to be associated with fewer prepared meals, while larger families have been associated with PMTH [53]. These findings align with the broader transition in Thailand toward convenience–oriented food acquisition, particularly in urban settings [37, 38] Rising incomes and higher disposable incomes drive the preference for convenience foods, prioritizing time savings [54–56]. In Bangkok, convenience is a key factor in food choices, with prepared meals being the most popular option [55]. Similar outcomes were reported in Vietnam that rising incomes drive transitions with affluent households preferring luxury foods like meat and milk and reducing their reliance on rice, a regional staple [57, 58]. Global retailers emerged a power engine of globalization and exerted huge impacts on food systems and environment with far reaching implications [59]. Ultra–processed foods showed the highest sales growth between 2012 and 2021 and are projected to continue increasing [60]. This food transition, closely tied to urbanization and economic development, is most evident among wealthier and younger populations and has contributed to rising NCD risks [28, 61, 62]. Urbanization and economic growth in Southeast Asia have driven dietary changes and the rise of fast–food chains and convenience foods [50, 63–68]. Younger generations prefer Western–style and ultra-processed foods, while older generation shows concern over declining diet quality [43–45]. Evidence from Thailand and neighboring countries suggests that multigenerational households maintain better dietary habits [41, 43, 46–49]. However, marketing and food regulations remain underexplored dimensions of the food environment in Thailand and Southeast Asia [49, 69–76].

Ultra–processed food sales in Asia rose significantly from 2000 to 2013, driven by market concentration and transnational influences in the grocery retail sector, key factors in the region’s nutrition transition [70]. Global change in lifestyle has changed the consumption of foods from home cooked to eating-out and convenience foods [77]. Busy life and overtime working push consumers to purchase prepared food to take home as they do not have time or skills to prepare meals [77–79]. Many developing countries faced this rapid shift in diet that also brought the epidemiological transition due to low physical activity and body composition [80]. Thailand is moving toward dietary sufficiency and with a moderate hunger level ranked 53rd out of 116 countries according to the 2023 Global Hunger Index (GHI). About 8.8% of Thailand’s population (approximately 6.2 million people) face insufficient nutrition [81]. Aging is major demographic factor found to have substantial impacts on food demand and expenditures [82, 83]. The household composition and family size are found to be the key factors to explain the differences in food consumption patterns in different age groups especially among elderly [84]. The elderly members of house tends to eat healthy food including vegetables, fish, and fruits [62]. However, the study was conducted only on the consumption of fresh food items. In Indonesia, teens often socialize at retail stores, drawn by the affordability of ready meals and convenience with microwaves and hot water available [85, 86]. Despite the increasing popularity of ready to eat meals among ASEAN countries including Thailand and Malaysia, consumers, especially working women, prefer ready–to–cook products due to concerns about preservatives and food coloring [87, 88]. In northeastern Thailand it is reported that young people consider the traditional food better [41]. In Laos, urbanization shapes dietary choices with mothers in central urban areas consuming more meat and milk but less fish and cereals than those in semi–urban regions [67, 89, 90]. Women in Singapore [44, 45], East Jakarta (Indonesia) [46], and Vientiane (Lao PDR) [89] cited busy work schedules and the affordability of prepared food as key reasons for eating out or buying ready–to–eat meals instead of cooking at home. In Singapore, women also reported using take–out as a strategy to balance differing family food preferences and accommodate intergenerational tastes [45].

**Fig. 7:**
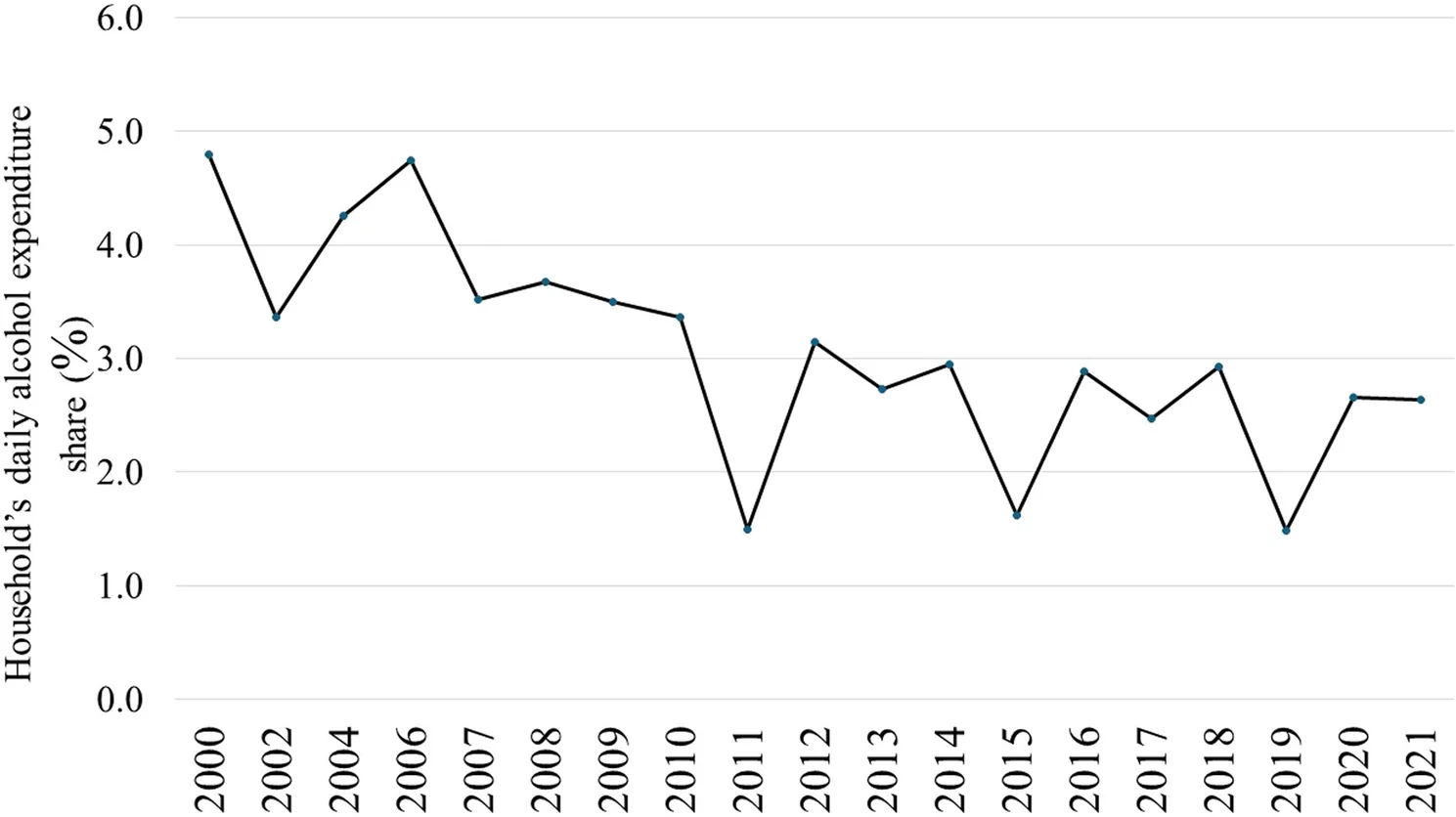
Percent alcohol consumption (at and away home) per Daily HH total food consumption expenditures

Alcohol/lifestyle-related dietary patterns (Spirits, SMEAH, and PF-Spirits) point out a lifestyle with social drinking norms in Thailand, where socializing and communal activities often involve alcohol [91, 92]. The percent share of alcohol-related expenses in Thai household food expenditures decreased from 4.8% in 2000 to 2.6% in 2021 (NSO data: Fig. 7). This does not necessarily indicate a reduction in health risks. The observed decline in household alcohol expenditure does not necessarily translate into a proportional reduction in health risks. This apparent paradox may be due to changes in consumption patterns rather than a true decrease in total intake. It is possible that people may substitute cheaper or unrecorded alcohol, and binge drinking may also continue. This helps explain why Thailand still ranked second in Southeast Asia for per capita alcohol consumption in 2019 [93], at 6.86 L, underscoring the need for ongoing interventions. Thailand has a relaxed cultural environment that promotes social drinking, as compared to Muslim countries like Malaysia [94] and Indonesia [95] where religious restrictions limit alcohol use. But Cambodia [96], Laos [97], Myanmar [98], and Vietnam [99, 100] have drinking patterns similar to those in Thailand. Compared to other ASEAN nations, Thailand has a relatively strong alcohol regulatory framework, the “Alcoholic Beverage Control Act”, which contrasts with weak enforcement and widespread informal alcohol markets in Vietnam [99] and Laos [97]. There are limited regulations and workplace-driven practices in Cambodia, which reinforce the high health risks associated with consumption. The ongoing health risks associated with alcohol are further worsened by its link to poor dietary choices, such as low fruit and vegetable intake and a preference for deep–fried foods. This pattern is strongly linked to cardiovascular and other health risks, even at moderate average consumption levels [92]. In Thailand, alcohol remains a major public health concern, contributing significantly to non-communicable diseases. It accounts for a substantial part of the national disease burden and economic costs.

These patterns (Fig. 4) were prevalent in the Northern and Northeastern regions, while BMR had exhibited a moderate prevalence that has been declining over time. It was also possible that the COVID–19 pandemic may have accelerated this decline in 2020 and 2021. However, the data from 2017 to 2018 also indicated a downward trend. This may suggest that the decline in adherence to these patterns was rooted in broader societal and cultural changes, rather than being a consequence of a pandemic. This pattern also showed a significant negative (*p* < 0.01) association with age, which indicates a trend of dining out and drinking among young generation specifically in BMR. Economically active members, their age, and monthly food consumption expenses were directly related to alcohol consumption.

The results of this study do not align with the previous studies in Thailand which reported that the alcohol consumption increases with age [101, 102]. Gender shows negative associations (*p* < 0.01), explaining that females (coded as 2) are significantly less likely to exhibit the alcohol pattern compared to males (coded as 1). Previous research based on Thailand NSO data highlighted that men were likely to be current drinkers compared to women [101, 102]. Larger households with more economically active members were positively associated (*p* < 0.01) with this diet (Table S6 and Table S7). Higher total incomes make it affordable and a way to socialize and relax. Conversely, higher household income was negatively associated with alcohol consumption, suggesting that increased earnings do not necessarily translate to increased drinking habits. In 2006, the health costs of alcohol consumption were estimated to be 2% of GDP [103, 104], which was decreased to 1.02% in 2021 [104], but it’s important to note that this decrease might be due to various factors, such as changes in alcohol consumption patterns, improved public health interventions, or economic factors. Association of smoking and drinking with dietary habits studied among Thai adolescents in BMR and other parts of the country concluded that smokers and alcohol drinkers were less likely to have healthy food consumption habits [101, 102, 105–107].

Alcohol/lifestyle related diets also included TTMHS pattern, which possess high consumption of sugars among Thai adults and the prevalence observed in and around the BMR region [108]. The availability of diverse food choices at affordable prices has shifted the diet of Thai people from being high in carbohydrates to one that includes more proteins and fats, with the consumption of added free sugars increasing each year [109]. A sedentary lifestyle, coupled with a diet low in fruits and vegetables and high in added sugars, is prevalent among Thai people, as reflected in the TTMHS (Table S7). The association analysis suggests that this pattern was significantly influenced by the education level of the household head, household income, and food expenditures, highlighting the importance of financial resources and educational background in adherence to this pattern. However, gender shows no significant contribution, while other variables, such as age and household size, exhibit negative associations. The negative association presents the idea of less sugar consumption in the elderly due to health reasons. The increase in free sugars with minimal nutritional value is filtering through to infants, children, and adolescents in the Thai population due to digital marketing. Sugar–sweetened beverages were the most commonly advertised food products on Free TV in Thailand, and baby/toddler milk formulae were mostly advertised on digital TV [75]. In Thailand, rising obesity and NCDs, including cardiovascular disease and diabetes, were linked to increased high–fat, sugar, and salt (HFSS) containing food consumption. Obesity rose from 37.5% in 2014 to 42.2% in 2019, with sugar intake increasing from 95 g/day to 125 g/day. Reducing consumption of HFSS foods can lower NCD risk. A review reported that the sugar intake among Thais remained unclear due to inconsistent studies and methodological limitations. A nationally representative survey with improved methods is needed to identify levels and sources of sugar consumption across population groups [110].

Meat dominant diets included patterns from disaggregated food data, including TTMM-FCP (highest loading for pork skin 0.40), MVM-FCP (pork 0.28 and chicken 0.22) MSF-FCoP (0.22 for chicken), MPF-FCoP (pork 0.44), and MVM-FCoP (chicken 0.30 and pork 0.28). Three out of five patterns were dominated by pork. These patterns exhibit an association with economically active household members and monthly food expenses, emphasizing the role of financial and working capacity in supporting protein-rich or seafood-inclusive diets. (Table S7). Researchers have also found that pork consumption in Thailand is related to the hepatitis E virus, and its prevalence was linked with other meat varieties too, that are sold in the fresh market of Bangkok [111, 112]. Also, studies for rural Thailand found that approximately 70% of S.suis infections investigated were associated with raw pork consumption [113, 114]. Other potential health risks associated with pork consumption include increased risk of heart disease due to its high saturated fat content, high levels of sodium in processed pork products can contribute to high blood pressure, and if it is not cooked properly, pork can contain parasites like Trichinella and Taenia *spp*, which cause trichinosis [115, 116] and taeniasis [117, 118] respectively. Similarly, in Myanmar, while the overall quantity of animal–source foods has not changed significantly, poultry consumption has increased substantially, reflecting shifts in dietary composition [119].

Seafood and fish have long been staples in the BMR and southern region (Fig. 4) due to their proximity to the coastlines and freshwater sources, ensuring high availability and affordability. The rich diversity of seafood products in local markets supports the development of various meal patterns, including Vegetables and Seafood Meals (Vege-Seafood), Seafood and Prepared Food (SPF), and Meat and Seafood Meals (MSM). In the BMR, urbanization and the wide availability of fresh and processed seafood encourage households to incorporate these ingredients into everyday meals, often blending traditional and modern culinary practices. Meanwhile, in Southern Thailand, the seafood culture was deeply embedded in local traditions. Many Southern dishes rely heavily on seafood combined with regional ingredients. People in these regions may favor seafood not only for its flavor but also for perceived health benefits. Dietary restrictions, whether they push individuals toward religious or health-related choices, may lead them to choose fish and seafood over other meats. In particular, the shift toward healthier eating has bolstered seafood consumption, driven by its lower fat content and high omega-3 fatty acid content, aligning with global health trends. But the excessive use of fish and seafood can also cause harm if they are contaminated with carcinogenic materials and microplastics. Several studies for coastal areas of Thailand reported the presence of such material in seafood that can be a health risk if consumed for a longer period [120–131]. In this study, the common factors from the association analysis that enable household access to seafood-rich diets were economically active members, monthly food consumption expenses, household income, as well as age and education of the household head. These findings align with a Thai study that highlighted the influence of economically active members and household income in enabling the purchase of seafood, while higher education levels are associated with greater awareness of the nutritional benefits of seafood [132].

The expenditure on foods in Thailand has also evolved over the time and faced a shift in food expenditures from home–prepared meals to ready–to–eat food, food delivery to home, and dine out options. Food accounted for a significant share of 33.7% of total household expenditures in 2015, increasing to 41.5% in 2021 to according (NSO data) [62]. The spending on food prepared at home declined from 64% in 2000 to 58.4% in 2021, while on prepared food or dining out was 36% which increased to 42% in 2021. This trend was more pronounced in BMR, where the expenditure on ready–to–eat food historically remained high, rising from 50% in 1990 [38] to 61% in 2000, and further increased to 67% by 2018. During the COVID–19 period (2019–2021), the expenditure on ready to eat food was stable between 60% and 64%. A similar trend was observed by another study in BMR and Chiangmai [133], showing a decline in average budget share for food prepared at home towards higher expenditure quartiles and households. Urbanization in Thailand has shifted household food consumption from traditional staple foods to higher–value and nutrient–rich options [12]. In rural areas, ready–to–eat food expenditures rose sharply from 12.2% in 1990 to 36.5% by 2021 [38]. Urban areas showed a more stable pattern with the proportion of home–prepared food versus ready–to–eat food shifting slightly from 52% to 48% in 2000 and 54% to 46% in 2021, respectively. Household dynamics also reflected this trend with male–headed households increasing their spending on ready–to–eat food from 35% in 2000 to 43% in 2021 while female–headed households saw a rise from 37% to 40% over the same period. This increase reflects a growing prioritization of food expenses, likely influenced by various factors such as inflation, changes in dietary habits, and economic conditions that affect household budgets. The shift could indicate a heightened focus on food security, as families allocate more of their resources towards meeting their nutritional needs, potentially in response to rising food prices and economic challenges. Understanding these trends is crucial for policymakers aiming to address food accessibility and security.

Marketing and regulations remain the least explored dimensions of the food environment, on consumer shopping behaviors and nutritional outcomes in Southeast Asia [49, 69–74] and Thailand [75, 76]. For instance, in Vietnam, sales of sugary carbonated beverages increased significantly after market liberalization compared to a control case such as Philippines. The trade and investment liberalization policies contributed to market domination by foreign transnational food companies [71]. Improving food education in Thailand requires an inclusive, practical approach rather than stereotyped models. Community–based intervention programs are urgently needed to combat malnutrition and the burden of NCDs. The government and related agencies must enforce stricter policies to regulate the frequency and content of on–air marketing of unhealthy foods, along with robust multi–sectoral governance [75, 76]. Encouraging dietary shifts such as reduced sugar, red meat, and saturated oil intake, while increasing consumption of vegetables and unsaturated oils can benefit both environmental sustainability and public health.

This research highlights cultural and regional dietary differences, such as the prominence of traditional Thai meals in rural areas and the adoption of mixed, convenience and meat focused diets in urban centers like the Bangkok Metropolitan Region. These findings provide actionable recommendations for policymakers, such as regulating unhealthy food advertising and promoting traditional diets, ensuring the study’s relevance for addressing public health concerns. Finally, the analysis contextualizes Thailand’s nutrition transition within broader global trends, offering a holistic perspective on the interplay between urbanization, economic development, and modern dietary shifts.

This study, despite its robust analysis, has several limitations. The study (i) solely relies on household food expenditure data rather than direct dietary intake, which do not reflect actual consumption or the diet quality and could miss nuances in food portions or preparation methods which influence diet quality; (ii) provides limited insight into the behavioral drivers of food consumption patterns, including individual preferences, cultural beliefs, or nutritional knowledge, critical for designing effective interventions; (iii) uses household survey data which introduces the potential for reporting biases for sensitive items like alcohol/spirits consumption, and this underreporting could skew results; (iv) temporal and regional consistency of the data might be impacted by changes in data collection methodologies over the years and may influence the comparability of findings spatiotemporally; and (v) this study focuses on Thailand and is valuable for understanding national trends, but findings may not be directly applicable to other Southeast Asian countries, despite regional dietary transitions.

The use of household food expenditure data as a proxy for dietary intake is a major limitation of this study, as expenditures do not directly reflect the quantity and nutritional quality of the food consumed. High expenditure for a given food category may reflect increased purchased quantities, higher prices, or the purchase of higher-quality food rather than greater intake. Likewise, expenditures on meals eaten away from home or convenience/prepared foods cannot be assumed to be unhealthy without additional information, including portion sizes and preparation methods. Consequently, the patterns identified in this study should be interpreted as household food purchasing strategies rather than precise patterns of households’ dietary intake. Meals eaten away from home and prepared meals often lack detailed ingredient-level information. Similarly, sensitive items such as alcohol may be underreported in household expenditure surveys, potentially biasing observed patterns. Future research is needed to combine household expenditure data with individual dietary intake surveys to improve calibration and validation of the analyses of expenditure-based dietary patterns. Further methodological limitations may arise from aggregating HSES data on foods into broad categories that combine foods with different nutritional values (e.g., vegetables grouped with starchy roots or tubers in some classifications), while others include heterogeneous items such as “spices and condiments” or “other prepared foods.” It’s difficult to assign precise nutritional interpretations to these broad groups based on specific food patterns. Some of the categories in disaggregated data remained difficult to interpret nutritionally, such as “breakfast,” “prepared foods,” and “other items.”

The nutritional relevance of most food groups can be contextualized using an internationally recognized dietary quality framework, such as the Global Diet Quality Score (GDQS), as well as dietary studies from Thailand. Some food groups are generally associated with positive diet quality, for example, fish and seafood, fruits and nuts, and vegetables, but sweets and sugars are mostly related to poor nutritional outcomes. The grains and cereals group in the HSES data mostly consists of rice and rice-based products, with limited consumption of whole grains. Health implications of oils and fats vary with their source, whether plant or animal, which contain different fatty acid profiles.

Food groups, such as meat and poultry, spices and condiments, and meals eaten outside the home, are also nutritionally diverse. In Thailand, street food and prepared meals often include processed meat, refined carbs, and high-sodium sauces, which is why the identified patterns should be seen as indicators of dietary habits rather than measures of diet quality. This analysis was done at the household level, and the results do not reflect individual members’ dietary intake. Patterns may not be able to present important differences among household members, such as by age or gender.

## Conclusions

The socio-demographic determinants of dietary intake in Southeast Asian countries like Thailand remain understudied. This study highlights the complexity and diversity of diets and food consumption patterns in Thailand over the past two decades, emphasizing shifts driven by urbanization, economic growth, and changing lifestyles. The changes in diets reflect evolving dietary preferences influenced by socioeconomic, demographic, and regional factors. While traditional meals and home cooking remain prevalent in rural, lower-income, and older households, urban and wealthier households show a stronger inclination towards mixed and convenience-oriented diets, such as prepared meals and dining out. The shift toward prepared meals and dining out underscores the growing reliance on convenience foods, particularly in metropolitan areas like Bangkok. The declining adherence to home-cooked meals and the rising consumption of ultra-processed and convenience foods pose significant public health challenges, including increased risks of non-communicable diseases (NCDs).

## Supplementary Information


Supplementary Material 1.



Supplementary Material 2.



Supplementary Material 3.



Supplementary Material 4.



Supplementary Material 5.



Supplementary Material 6: Table S1: HH expenditures on 13 food groups and specific foods items under each food groups for three years: 2011, 2015 and 2019. Table S2: Comparison of factor loadings for retained household food consumption patterns generated using aggregated (2000–2021) food data and those generated within specific years and regions. Table S3: Comparison of factor loadings for retained household food consumption patterns generated using disaggregated (2011–2019) food data and those generated within specific years and regions. Table S4: Factor loadings for retained household food consumption and composition patterns generated using disaggregated (2011, 2015 & 2019) food data. Table S5: Factor loadings for retained household food consumption and composition patterns generated using aggregated (2011 & 2019) food data. Table S6: Regression model for association between Food Consumption Pattern–FCPs and Household characteristics, 2000–2021. Table S7: Regression model for association between Food Consumption/Composition Pattern–FCPs and Household characteristics, 2011, 2015 and 2019. Table S8: Daily household food expenditure shares (%) for overall Thailand and its five regions for the year 2021.


## Data Availability

Data is provided within the manuscript or supplementary information files. Data can be shared on demand.

## Abbreviations

BMR: Bangkok Metropolitan Region
FCoP: Food Composition Pattern
FCP: Food Consumption Pattern
GLM: Generalized Linear Model
HDDI: Household Dietary Diversity Index
HH: Household
HSES: Household Socio Economic Survey
MEAH: Meals Eaten Away Home
MEAH-PF: MEAH and Prepared Food
MPH: Meals Prepared at Home
MPF: Meat and Prepared Food
MSM: Meat and Seafood Meals
MVM: Meat and Vegetable Meals
NCD: Non–Communicable Disease
NSO: National Statistical Office
PCA: Principal Component Analysis
PC: Principal Component
PF-Spirits: Prepared Food and Spirits
PMTH: Prepared Meals Taken Home
SMEAH: Spirits and Meals Eaten Away Home
SPF: Seafood and Prepared Foods
TCPF: Traditional Condiment and Prepared Food
THB: Thai Baht
TTM: Traditional Thai Meals
TTMM: Traditional Thai Meat Meals
TTMHS: Traditional Thai Meals with High Sugar
VSF/Vege-Seafood: Vegetables and Seafood
