# Supplementary material for: Exploratory household food consumption patterns and their associations with socioeconomic characteristics in Thailand: analysis of 19 rounds of the national household socio – economic survey from 2000 to 2021
Source: Nutr J. 2026 Jul 7;25:96. doi: 10.1186/s12937-026-01353-3 (PMC13536753; doi:10.1186/s12937-026-01353-3)
Supplement: Supplementary file 1 [file 12937_2026_1353_MOESM1_ESM.docx]

| **Classification of Individual Food Items into Major Food Groups Used for HDDI Calculation (2011, 2015, 2019)** | |
| --- | --- |
|  |  |
| **Food Group** | **Included Food Items** |
| **Cereals and cereal products** | Rice; Glutinous rice; Bread; Chinese noodles/egg noodles; Thai vermicelli; Macaroni and spaghetti; Rice flour; Wheat, tapioca, and other flours; Curly rice noodles; Clear noodles; Noodles; Vermicelli; Breakfast cereals |
| **Vegetables** | Cabbage; Cauliflower; Choy; Mimosa; Cucumber; Chinese cabbage; Lettuce; Chinese broccoli; Kale; Mushroom; Chinese water spinach; Coriander; Long eggplant; Eggplant; Tomato; String beans; Gourd; Angled loofah; Raw papaya; Winter melon; Pumpkin; Lemon; Chili pepper; Celery; Spring onion; Green peas; Mung beans; Carrot; Bean sprouts; Culinary herbs; Tum leung; Chive flowers; Lemongrass; Ivy gourd; Chinese leek; Chinese gourd; Baby corn; Asparagus; Sweet corn; Parsley/Culantro; Acacia pennata; Broccoli; Other fresh vegetables |
| **Fruits** | Banana; Orange; Papaya; Pineapple; Rambutan; Mango; Watermelon; Durian; Grapes; Apple; Guava; Rose apple; Pomelo; Longan; Mangosteen; Longkong; Lychee; Dragon fruit; Young coconut; Cantaloupe; Chinese pear; Other fruits; Preserved fruits; Canned fruits |
| **Meat and meat products** | Pork; Pork ribs; Other parts of pig; Beef, cow, or buffalo meat; Other parts of beef; Fresh chicken; Fresh chicken including organs; Sausages; Chinese sausage; Other fresh or cooked meats; Dried shredded pork; Sour pork or beef sausage |
| **Fish and seafood** | Grouper; Snakehead; Catfish; Nile tilapia; Carp; Cotton fish; Tuna; Tilapia; Pomfret (black or silver); Red snapper/giant seaperch; Sheatfish; Chub mackerel; Indian mackerel; Shrimp; Giant freshwater prawn; Whiteleg shrimp; Banana prawn; Giant tiger prawn; Squid; Blood cockle; Baby clam; Green mussel; Sea crab/blue crab; Blue swimming crab; Frog; Dried squid; Dried shrimp; Fish balls; Crab sticks |
| **Eggs** | Hen eggs; Duck eggs; Salted eggs; Other eggs |
| **Pulses, legumes, and nuts** | Soybean curd (tofu); Green chickpeas; Groundnuts; Peanuts; Other nuts |
| **Milk and dairy products** | Fresh milk; Condensed milk; Powdered milk; Cheese; Sour cream; Soybean milk |
| **Oils and fats** | Vegetable oil; Lard; Butter; Coconut cream; Margarine; Other oils and fats |
| **Sugar and sweets** | Candy; Ice cream; Desserts; Jelly and chocolate; White sugar; Palm sugar; Coconut sugar; Jam; Honey |
| **Condiments and seasonings** | Sauce; Cayenne pepper; Seasoning sauce; Fermented soybeans; Soybean paste; Salt; Fish sauce; Soy sauce; Oyster sauce; Chili sauce; Tomato sauce; Vinegar; Shrimp paste; Tamarind; Curry paste; Seasonings; Monosodium glutamate |
| **Beverages** | Coffee; Tea; Cocoa or chocolate drinks; Soda; Carbonated water; Vegetable and fruit juices; Smoothies or squash drinks; Drinking water; Soft drinks; Iced coffee; Caffeinated drinks; Prepared coffee; Prepared tea |
| **Prepared and convenience foods** | Rice and curry (packed rice); Fried rice; Stir-fried noodles (Rad Na/Pad See Ew); Red pork or crispy pork on rice; Prepared food dishes; Noodles; Hamburger or sandwich; Food ordered by phone or internet; Canned food; Semi-prepared foods |
