# Supplementary material for: Exploratory household food consumption patterns and their associations with socioeconomic characteristics in Thailand: analysis of 19 rounds of the national household socio – economic survey from 2000 to 2021
Source: Nutr J. 2026 Jul 7;25:96. doi: 10.1186/s12937-026-01353-3 (PMC13536753; doi:10.1186/s12937-026-01353-3)
Supplement: Supplementary file 2 [file 12937_2026_1353_MOESM2_ESM.docx]

**Thailand Household Socio-Economic Survey (HSES)**

The Thailand Household Socio-Economic Survey (HSES), conducted by the National Statistical Office (NSO), is the primary nationally representative household survey for monitoring socio-economic conditions, including income, expenditures, employment, and living standards. Historically, the HSES has used a stratified multi-stage sampling design with provinces (changwats) and urban/rural classification forming the primary strata, and sample enumeration districts or blocks/villages selected with probability proportional to size, followed by systematic selection of households within those units as the ultimate sampling units. In earlier rounds (e.g., 2000 and 2002), the survey employed a stratified two-stage design, with blocks in municipal areas and villages in non-municipal areas as primary sampling units and private households as secondary units, and sample sizes such as 46,600 households in 2002 with a national response rate of approximately 74.7%. More recent rounds (post-2010) continue annual national coverage, with the sample each year divided into twelve sub-samples surveyed on a rotating monthly basis, and annual samples on the order of tens of thousands of households (e.g., about 55,854 in 2019), all interviewed via face-to-face enumeration of household heads or designated members. Although publicly available documentation on later rounds does not consistently publish detailed response rates or precise nominal sample sizes for each cycle, these are recorded in NSO technical reports and microdata metadata. For a future online supplement, tabulated information including survey year, sample size (households and individuals), questionnaire modules used (e.g., core socio-economic, expenditure, income), sampling strata and stages, and response rates should be compiled from NSO annual reports and data dictionaries. This supplement would enhance transparency and allow users and researchers to compare coverage and fieldwork characteristics across the 2000–2021 period. International repositories such as the ILO survey catalog provide additional metadata on individual survey rounds and can support these compilations where NSO documentation is limited (1)

| **Survey Year** | **Survey Frequency** | **Sampling Design** | **Sampling Strata** | **Primary Sampling Units (PSUs)** | **Secondary Units (Households)** | **Target Sample Size (HHs)** | **Completed Sample Size (HHs)** | **Response Rate (%)** | **Questionnaire Modules Included** | **Mode of Data Collection** | **Reference / Technical Report** |
| --- | --- | --- | --- | --- | --- | --- | --- | --- | --- | --- | --- |
| 2000 | Biennial | Stratified multi-stage | Province × Urban/Rural | Blocks (urban), Villages (rural) | Private households |  | 24747 |  | Core SES, Income, Expenditure | Face-to-face | NSO HSES 2000 |
| 2002 | Biennial | Stratified two-stage | Province × Urban/Rural | Blocks/Villages | Private households | 46,600 | 34785 | ~74.7 | Core SES, Income, Expenditure | Face-to-face | NSO HSES 2002 |
| 2004 | Biennial | Stratified multi-stage | Province × Urban/Rural | Blocks/Villages | Private households |  | 43055 |  | Core SES, Income, Expenditure | Face-to-face | NSO HSES 2004 |
| 2010 | Annual | Stratified multi-stage | Province × Urban/Rural | Enumeration areas | Private households |  | 44273 |  | Core SES, Income, Expenditure | Face-to-face | NSO HSES 2010 |
| 2015 | Annual | Stratified multi-stage | Province × Urban/Rural | Enumeration areas | Private households |  | 43400 |  | Core SES, Income, Expenditure | Face-to-face | NSO HSES 2015 |
| 2019 | Annual | Stratified multi-stage | Province × Urban/Rural | Enumeration areas | Private households | ~55,854 | 45586 |  | Core SES, Income, Expenditure | Face-to-face | NSO HSES 2019 |
| 2020 | Annual | Stratified multi-stage | Province × Urban/Rural | Enumeration areas | Private households |  | 48210 |  | Core SES, Income, Expenditure | Face-to-face (COVID-adjusted) | NSO HSES 2020 |
| 2021 | Annual | Stratified multi-stage | Province × Urban/Rural | Enumeration areas | Private households |  | 46840 |  | Core SES, Income, Expenditure | Face-to-face | NSO HSES 2021 |
| 2022 | Annual | Stratified multi-stage | Province × Urban/Rural | Enumeration areas | Private households |  | 50277 |  | Core SES, Income, Expenditure | Face-to-face | NSO HSES 2022 |
| 2023 | Annual | Stratified multi-stage | Province × Urban/Rural | Enumeration areas | Private households |  | 49664 |  | Core SES, Income, Expenditure | Face-to-face | NSO HSES 2023 |
| 2024 | Annual | Stratified multi-stage | Province × Urban/Rural | Enumeration areas | Private households |  | 51055 |  | Core SES, Income, Expenditure | Face-to-face | NSO HSES 2024 |

Reference

1.	NSO. The Household Socio-Economic Survey [Internet]. 2025. Available from: https://www.nso.go.th/nsoweb/nso/survey_detail/qC?set_lang=en#gsc.tab=0
