# Supplementary material for: Exploratory household food consumption patterns and their associations with socioeconomic characteristics in Thailand: analysis of 19 rounds of the national household socio – economic survey from 2000 to 2021
Source: Nutr J. 2026 Jul 7;25:96. doi: 10.1186/s12937-026-01353-3 (PMC13536753; doi:10.1186/s12937-026-01353-3)
Supplement: Supplementary file 3 [file 12937_2026_1353_MOESM3_ESM.docx]

Scatter plots for HDDI vs Pattern adherence 2000-2021

Scatter plots for HDDI vs Pattern adherence 2011, 2015, 2019
