# Supplementary material for: Exploratory household food consumption patterns and their associations with socioeconomic characteristics in Thailand: analysis of 19 rounds of the national household socio – economic survey from 2000 to 2021
Source: Nutr J. 2026 Jul 7;25:96. doi: 10.1186/s12937-026-01353-3 (PMC13536753; doi:10.1186/s12937-026-01353-3)
Supplement: Supplementary file 4 [file 12937_2026_1353_MOESM4_ESM.docx]

Grains and Cereal

|  | % Expense |
| --- | --- |
| 2000 | 15.8558 |
| 2002 | 12.648 |
| 2004 | 12.9245 |
| 2006 | 12.8935 |
| 2007 | 13.3875 |
| 2008 | 14.3759 |
| 2009 | 13.6037 |
| 2010 | 13.8447 |
| 2011 | 14.4003 |
| 2012 | 14.5481 |
| 2013 | 14.0827 |
| 2014 | 13.5266 |
| 2015 | 12.5269 |
| 2016 | 12.6294 |
| 2017 | 10.9889 |
| 2018 | 11.6905 |
| 2019 | 11.5245 |
| 2020 | 12.9853 |
| 2021 | 11.9182 |

Meat and Poultry

|  |  |
| --- | --- |
| 2000 | 10.3716 |
| 2002 | 11.6941 |
| 2004 | 9.58376 |
| 2006 | 9.9362 |
| 2007 | 10.2008 |
| 2008 | 9.40883 |
| 2009 | 9.62655 |
| 2010 | 9.47674 |
| 2011 | 13.3111 |
| 2012 | 11.3718 |
| 2013 | 10.8062 |
| 2014 | 10.7249 |
| 2015 | 12.6965 |
| 2016 | 9.42592 |
| 2017 | 8.44477 |
| 2018 | 8.92382 |
| 2019 | 12.7492 |
| 2020 | 10.6365 |
| 2021 | 10.8856 |

Fish and Seafood

|  | % Expense |
| --- | --- |
| 2000 | 8.73405 |
| 2002 | 9.58184 |
| 2004 | 7.82779 |
| 2006 | 8.16865 |
| 2007 | 8.50299 |
| 2008 | 7.85968 |
| 2009 | 8.02108 |
| 2010 | 7.93295 |
| 2011 | 9.61054 |
| 2012 | 8.01468 |
| 2013 | 7.85877 |
| 2014 | 8.01585 |
| 2015 | 9.54196 |
| 2016 | 8.21667 |
| 2017 | 7.87028 |
| 2018 | 8.37854 |
| 2019 | 10.0459 |
| 2020 | 9.2638 |
| 2021 | 9.15072 |

Milk, Cheese and Eggs

|  |  |
| --- | --- |
| 2000 | 6.83879 |
| 2002 | 6.66587 |
| 2004 | 6.88518 |
| 2006 | 7.47525 |
| 2007 | 6.9355 |
| 2008 | 6.58885 |
| 2009 | 6.3926 |
| 2010 | 6.17838 |
| 2011 | 5.72088 |
| 2012 | 6.38446 |
| 2013 | 6.12524 |
| 2014 | 6.36931 |
| 2015 | 5.28621 |
| 2016 | 5.99409 |
| 2017 | 5.56155 |
| 2018 | 6.03603 |
| 2019 | 5.02232 |
| 2020 | 1.68686 |
| 2021 | 6.02706 |

Oil Fats

|  | % Expense |
| --- | --- |
| 2000 | 2.33053 |
| 2002 | 1.33666 |
| 2004 | 1.76889 |
| 2006 | 1.72716 |
| 2007 | 1.43105 |
| 2008 | 1.50357 |
| 2009 | 1.35317 |
| 2010 | 1.26009 |
| 2011 | 1.49388 |
| 2012 | 1.36845 |
| 2013 | 1.28458 |
| 2014 | 1.29467 |
| 2015 | 1.2166 |
| 2016 | 1.1611 |
| 2017 | 1.09692 |
| 2018 | 1.15006 |
| 2019 | 1.14389 |
| 2020 | 1.26912 |
| 2021 | 1.36958 |

Fruits and Nuts

|  |  |
| --- | --- |
| 2000 | 7.38626 |
| 2002 | 6.62784 |
| 2004 | 7.53679 |
| 2006 | 6.75209 |
| 2007 | 6.40762 |
| 2008 | 6.19122 |
| 2009 | 6.22704 |
| 2010 | 6.06708 |
| 2011 | 5.18659 |
| 2012 | 5.85136 |
| 2013 | 5.73353 |
| 2014 | 5.72952 |
| 2015 | 6.0076 |
| 2016 | 6.44117 |
| 2017 | 6.08427 |
| 2018 | 6.39122 |
| 2019 | 5.84799 |
| 2020 | 6.915 |
| 2021 | 6.78194 |

Vegetables

|  | % Expense |
| --- | --- |
| 2000 | 6.33633 |
| 2002 | 7.08642 |
| 2004 | 5.51426 |
| 2006 | 5.4652 |
| 2007 | 6.22584 |
| 2008 | 5.85468 |
| 2009 | 5.94828 |
| 2010 | 6.0691 |
| 2011 | 7.95417 |
| 2012 | 6.14083 |
| 2013 | 6.28317 |
| 2014 | 6.18213 |
| 2015 | 8.23681 |
| 2016 | 6.71325 |
| 2017 | 6.23892 |
| 2018 | 6.40422 |
| 2019 | 8.50493 |
| 2020 | 7.07183 |
| 2021 | 6.65961 |

Sugar and Sweets

|  |  |
| --- | --- |
| 2000 | 3.31352 |
| 2002 | 2.9596 |
| 2004 | 3.14893 |
| 2006 | 3.91612 |
| 2007 | 3.83213 |
| 2008 | 3.80095 |
| 2009 | 3.83714 |
| 2010 | 3.86476 |
| 2011 | 2.58006 |
| 2012 | 2.91998 |
| 2013 | 2.70329 |
| 2014 | 2.61804 |
| 2015 | 2.55262 |
| 2016 | 3.42496 |
| 2017 | 3.10261 |
| 2018 | 3.26022 |
| 2019 | 2.2988 |
| 2020 | 3.64582 |
| 2021 | 3.46119 |

Spices and Condiment

|  | % Expense |
| --- | --- |
| 2000 | 3.11349 |
| 2002 | 4.373 |
| 2004 | 4.3829 |
| 2006 | 1.76855 |
| 2007 | 2.36181 |
| 2008 | 2.02432 |
| 2009 | 1.95841 |
| 2010 | 1.90262 |
| 2011 | 2.14375 |
| 2012 | 1.7494 |
| 2013 | 1.75659 |
| 2014 | 1.80916 |
| 2015 | 2.0238 |
| 2016 | 1.81102 |
| 2017 | 1.7595 |
| 2018 | 1.97137 |
| 2019 | 2.12554 |
| 2020 | 2.12538 |
| 2021 | 2.09789 |

Prepared Meals Taken Home (PMTH)

|  |  |
| --- | --- |
| 2000 | 11.5164 |
| 2002 | 14.7053 |
| 2004 | 14.8271 |
| 2006 | 14.7295 |
| 2007 | 16.0762 |
| 2008 | 15.9812 |
| 2009 | 16.3898 |
| 2010 | 17.0868 |
| 2011 | 20.6801 |
| 2012 | 17.3044 |
| 2013 | 18.0638 |
| 2014 | 18.1201 |
| 2015 | 19.9716 |
| 2016 | 15.631 |
| 2017 | 13.0503 |
| 2018 | 16.6503 |
| 2019 | 20.3371 |
| 2020 | 16.7258 |
| 2021 | 16.8576 |

Non-Alcoholic Beverage (at Home)

|  | % Expense |
| --- | --- |
| 2000 | 2.56139 |
| 2002 | 2.58839 |
| 2004 | 2.89123 |
| 2006 | 5.09219 |
| 2007 | 4.23189 |
| 2008 | 3.97111 |
| 2009 | 4.1231 |
| 2010 | 4.36814 |
| 2011 | 4.42674 |
| 2012 | 3.80956 |
| 2013 | 4.10409 |
| 2014 | 4.4437 |
| 2015 | 4.76286 |
| 2016 | 5.44336 |
| 2017 | 14.4326 |
| 2018 | 5.61199 |
| 2019 | 5.32762 |
| 2020 | 6.65488 |
| 2021 | 6.70132 |

Alcohol Consumption (at and away home)

|  | % Expense |
| --- | --- |
| 2000 | 4.79477 |
| 2002 | 3.36094 |
| 2004 | 4.25052 |
| 2006 | 4.74237 |
| 2007 | 3.51839 |
| 2008 | 3.67923 |
| 2009 | 3.49248 |
| 2010 | 3.36265 |
| 2011 | 1.49271 |
| 2012 | 3.1416 |
| 2013 | 2.73472 |
| 2014 | 2.94503 |
| 2015 | 1.61969 |
| 2016 | 2.88853 |
| 2017 | 2.46572 |
| 2018 | 2.92376 |
| 2019 | 1.48396 |
| 2020 | 2.65783 |
| 2021 | 2.63767 |

Meals Eaten Away Home (MEAH)

|  | % Expense |
| --- | --- |
| 2000 | 16.8471 |
| 2002 | 16.372 |
| 2004 | 18.4582 |
| 2006 | 17.322 |
| 2007 | 16.879 |
| 2008 | 18.756 |
| 2009 | 19.0129 |
| 2010 | 18.5769 |
| 2011 | 10.8994 |
| 2012 | 17.3954 |
| 2013 | 18.454 |
| 2014 | 18.2142 |
| 2015 | 13.4302 |
| 2016 | 20.2059 |
| 2017 | 18.8875 |
| 2018 | 20.5788 |
| 2019 | 13.4676 |
| 2020 | 18.3578 |
| 2021 | 15.426 |
