# Supplementary material for: Exploratory household food consumption patterns and their associations with socioeconomic characteristics in Thailand: analysis of 19 rounds of the national household socio – economic survey from 2000 to 2021
Source: Nutr J. 2026 Jul 7;25:96. doi: 10.1186/s12937-026-01353-3 (PMC13536753; doi:10.1186/s12937-026-01353-3)
Supplement: Supplementary file 5 [file 12937_2026_1353_MOESM5_ESM.docx]

## 1.1. Adherence of food patterns to HH characteristics

Table 1 and Table 2 presents household (HH) characteristics with corresponding data on food consumption patterns across various periods, regions, and socioeconomic categories. The data for 2011, 2015 and 2019 were split into aggregated and disaggregated components.

1.1.1. Year

FCPs 2000–2021: The mixed pattern (PC1) exhibited fluctuations over the years (Table 2), with adherence decreasing from 4.8% in 2000 to 1.3% in 2006, then stabilizing around 2.0 in later years. MPH (PC2) and Spirits (PC3) remained relatively stable over the years, indicating that these household patterns have not changed significantly.

FCoPs 2000–2021: In 2000, households have the highest MPH (PC1) values (Table 2), indicating greater diversity in food consumption. However, over time, PC1 slightly decreased, while Spirits, MEAH (PC2), and PMTH (PC3) remained relatively stable.

FCPs 2011, 2015 and 2019: PF-Spirits (PC1) and MVM (PC4) from disaggregated data declined, while the adherence for TTMM (PC2) significantly increase from 49% to 61%. Adherence for TTM (PC4) also increased a little (Table 2). The FCPs from aggregated data showed little decrease in MP (PC1) and SFPF (PC4) and a slight increase TTM (PC2) and SCM (PC3) in 2019 compared to 2011.

FCoPs 2011, 2015 and 2019: TTMHS (PC1) and MPF (PC2) from disaggregated data increased over the years while MVM (PC3) and MSM (PC4) decreased. FCoPs from aggregated data showed increase for a combo of MEAH-PF (PC1), and TCPF (PC3) was increasing while TTM (PC2), and MEAH (PC4) showed reduction in adherence (Table 2).

1.1.2. Regions

FCP 2000–2021: The most urbanized and tourism–centered regions, particularly the Bangkok Metropolitan Region (BMR) and the South, exhibit the highest adherence to the mixed pattern (PC1 at 2.38 and 2.41, respectively). This pattern represents a mix of Meals Prepared at Home (MPH), Meals Eaten Away from Home (MEAH), and Prepared Meals Taken Home (PMTH), reflecting the diverse nature of these regions due to tourism and a blend of local and international influences. However, adherence to MPH (PC2) and Spirits (PC3) remained relatively consistent across all regions, with only minor variations.

FCoP 2000–2021: Households in the North and Northeast regions show the highest adherence to MPH (PC1), while BMR has the lowest adherence to MPH (PC1) but the highest values for spirits and MEAH (PC2). The Northeastern region showed highest adherence to PMTH (PC3).

FCP 2011, 2015 and 2019: Adherence to PF and Spirits is surprisingly lowest in BMR (20%) and highest in Northern and Northeastern region (28.5 and 29%) for disaggregated data. However, the southern region exhibits the lowest values for all other PCs. For aggregated data mixed pattern has the lowest values (7.3) for Northern and Northeastern region but highest (up to 59.4) for TTM (similar trend in TTM from disaggregated). The adherence for VSF and SFPF remained highest in BMR (79 and 58% respectively).

FCoP 2011, 2015 and 2019: BMR exhibits the highest adherence to TTMHS (80%) while Northern and Northeastern regions remained at the lowest (up to 71.5). Southern region provinces show high value (75%) for TTMHS but remained lowest for the rest of the patterns from disaggregated data.

MEAH–PF from aggregated data have high values for the BMR, central and southern regions (81–84%). From aggregated data TTM has low adherence in Northern and Northeastern regions but shows higher values for BMR, central, and Southern regions (25–25.6%). The TCPF remained prominent in northeast and MEAH in Northern and Northeastern regions.

1.1.3. Communities

FCP 2000–2021: Relatively higher values for the mixed pattern (PC1–2.16) were observed in rural areas compared to urban areas (2.1). There were minimal differences between urban and rural communities for MPH (PC2) and Spirits (PC3), indicating that these FCPs are consistent across both communities.

FCoP 2000–2021: Rural households exhibited higher adherence to MPH (PC1–14.38), Spirits, and MEAH (PC2–50.57) than urban households (14.4 and 49.8, respectively), while urban households showed higher values for PMTH (PC3).

FCP 2011, 2015 and 2019: Urban households tended to have lower adherence to PF and Spirits (22.4%) while higher for rural households (28%). Adherence for TTM almost remained similar for rural and urban but TTMM and MVM remain prevalent in urban communities. Values in FCPs derived from disaggregated data, mixed pattern, VSF, and SFPF remained high in urban communities and TTM in rural households.

FCoP 2011, 2015 and 2019: Urban households had lower adherence to TTMHS, MVM, and MSM while MPF remained low in rural areas from the disaggregated data. Patterns from the aggregated data for urban households had higher MEAH–PF and TCPF and lower adherence to the rest of the patterns compared to rural households.

1.1.4. HH Head Gender

FCP 2000–2021: Male–headed households show slightly higher MPH (PC1–2.08) values compared to female–headed households (1.84). MPH (PC2), Spirits, and MEAH (PC3) remained consistent across genders, indicating no significant differences.

FCoP 2000–2021: Male–headed households exhibit slightly higher adherence to the three patterns compared to female–headed households.

FCP 2011, 2015 and 2019: Female–headed households showed slightly higher adherence to PF, Spirits, and TTMM, while TTM and MVM remained high for male-headed households from disaggregated data. The FCPs from aggregated data had slightly higher values for mixed pattern and TTM among male–headed households, while VSF and SFPF were higher for female-headed households, however differences were minimum.

FCoP 2011, 2015 and 2019: The adherence for both genders in FCoP from disaggregated and aggregated data remained almost similar with slight differences.

1.1.5. HH Head Age

FCP 2000–2021: Low adherence to the mixed pattern (PC1–1.46) and MPH (PC2–53) is observed among younger household heads (9–25 years), with adherence increasing as the age of the household head rises. Spirits (PC3) show consistency across all age groups, indicating that age–related differences do not significantly affect this pattern.

FCoP 2000–2021: A similar increasing trend for MPH with respect to household head age was observed. The oldest household heads (66–100 years) exhibit higher MPH (PC1–17.66) and PMTH (79.9), while younger household heads (9–25 years) showed high values for Spirits and MEAH (50.59).

FCP 2011, 2015 and 2019: FCPs derived from disaggregated data showed highest adherence to PF and Spirits patterns among household heads aged 56 years or above (up to 28.3%). The TTMM pattern remained similar across all age groups with slight variations, but adherence to TTM pattern increased with the increase in age of household head. The MVM pattern was more prevalent among younger household heads. The mixed pattern showed higher adherence among household heads aged from 25 to 65 years (8.3–8.7%), the age group associated with the workforce. The TTM pattern also showed an increasing trend with age, while the VSF and SFPF patterns were consistent across all the age groups with slight variations and no specific trend in the aggregated data.

FCoP 2011, 2015 and 2019: FCPs derived from disaggregated data revealed that the younger household heads (aged 9–25 years) have significantly higher adherence to the TTMHS pattern compared to older groups. The other three PCs, including MPF, MVM, and MSM showed consistent adherence with slight variations. FCPs from aggregated data indicated that the MEAH-PF and MEAH patterns were more prominent among younger household heads (aged 9–25 years) and were less prominent among older groups. The TTM and TCPF patterns were consistent across all age groups with slight variations.

1.1.6. HH Head Education

FCP 2000 – 2021: The mixed pattern showed variation across education levels, with extreme values in categories like informal education (4.34), vocational education (4.05), and occupational education (5.73). MPH and Spirits vary within a certain range but were not significantly different, indicating that education levels do not heavily impact these FCPs.

FCoP 2000–2021: Households where the head has informal and occupational education levels have highest adherence to MPH and PMTH, but the heads with vacation education found to be of higher adherence (51%) to Spirits and MEAH.

FCP 2011, 2015 and 2019: Disaggregated data show that the adherence to the PF and Spirits pattern increases with the education level of household heads, peaking among those with occupational education. The highest adherence (28.3%) was found in households headed by individuals with preprimary and primary education. The TTMM pattern showed no clear trend but had the highest adherence among doctoral degree holders (58%). Both TTM and MVM patterns displayed no distinct trends, with values remaining within a consistent range and slight variations across educational groups for FCPs. Aggregated data indicate that the mixed pattern is most prominent among household heads with master's and doctoral degrees (10–12%) and lowest (3.6%) among those with vocational education. TTM adherence was highest for heads with preprimary and primary education, as well as those with occupational degrees (58.31–58.86%). VSF and SFPF patterns were common among doctoral degree holders with adherence remaining consistent across other educational groups and showing minor variations.

FCoP 2011, 2015 and 2019: Disaggregated data show that the TTMHS pattern remains prominent among households led by heads with education levels ranging from vocational to university degrees (80–85%). MPF and MVM adherence was consistent across all education groups, showing slight variations and no specific trends. MSM adherence was higher among heads with lower education levels (preprimary, primary, and secondary education) and those with professional training (46.4–48.4%). Aggregated data indicate that households with heads holding vocational to university degrees (including doctoral degrees) have the highest adherence to MEAH-PF (86–91%). TCPF and MEAH patterns were most prominent among those with vocational education, while TTM adherence remained consistent across all groups, with minor variations.

1.1.7. Number of Economically Active Members

FCP 2000–2021: Values for the mixed pattern (PC1) increase with the number of economically active members, rising from 1.35 (0 members) to 6.59 (9–14 members). The other PCs were within a certain range of values.

FCoP 2000–2021: Households with more economically active members (3–5 and 9–14 members) showed higher values for MPH (PC1) at 16.75 and 16.57, respectively. Spirits and MEAH (PC2) increased as the number of economically active members increased, while households with 0 or very few economically active members show lower PC1 and higher PC3 values.

FCP 2011, 2015 and 2019: FCPs from disaggregated data indicate that households with 1–2 or 9–14 economically active members have the lowest adherence to the prepared food and Spirits pattern. Adherence to the TTMM pattern decreased as the number of active members increased, while TTM showed an increasing trend in adherence with more members (ranging from 71.2% to 72.5%), except for the largest group, which had the lowest adherence (69%). MVM do not display a clear trend, but the value remained high for households with 9–14 active members (48.2%). FCPs from aggregated data showed high adherence to the mixed pattern among households with more than three active members. TTM and SFPF showed consistent values across all groups, with minimal variations.

FCoP 2011, 2015 and 2019: FCoPs from disaggregated reveal that adherence to the MPF and MVM patterns decreased as the number of active members increased, while TTMHS and MSM values were relatively stable, showing only minor variations. Aggregated data highlight the highest adherence to MEAH-PF among households with 1–2 and 9–14 members, and the highest adherence to TTM for households with 9–14 active members. TCPF and MEAH adherence were consistent across all groups, with minor variations.

1.1.8. Number of Household Members

FCP 2000–2021: Adherence to the mixed pattern (PC1–1.68) increased with the household size, indicating that larger households have distinct primary characteristics compared to smaller ones. PC2 and PC3 were consistent with slight variations across household sizes.

FCoP 2000–2021: Larger households with 7–10 members showed higher adherence to MPH (PC1–16.84), while households with 11–15 members displayed lower adherence (PC1–16.16) but higher Spirits and MEAH (PC2 – 541.21). Adherence to PMTH (PC3) decreased as the number of household members increased.

FCP 2011, 2015 and 2019: Disaggregated data reveal that adherence to the PF and Spirits pattern increased as the number of household members increase, while adherence to the TTMM and MVM patterns decreased with larger household sizes. TTM adherence remained within a certain range, showing no specific trend. Aggregated data indicated that adherence to mixed patterns, TTM, and SFPF increased with household size, while VFS adherence decreased as household size grew.

FCoP 2011, 2015 and 2019: Disaggregated data showed that the adherence to TTMHS, MPF, and MSM patterns decreased as household size increased, while MVM adherence were within a certain range, with no specific trend. Aggregated data highlights that households with 0 and 9–14 members exhibit higher adherence to MEAH–PF (up to 81.3%) than other groups (PC1–71.5% and –70.85%, respectively). Adherence to TTM, TCPF, and MEAH patterns decreased as household size increased.

1.1.9. Household Income

FCP 2000–2021: Adherence to the mixed pattern (PC1) increased with income levels, peaking in the higher income brackets (≥400,000 Baht at 4.14). MPH (PC2) showed a slight decline at higher income levels. Spirits (PC3) also showed a declining trend towards higher incomes (200,001–400,000 Baht at 21.27), but it increased again for those earning ≥400,000 Baht, reaching 21.48.

FCoP 2000–2021: Households with incomes of 1–10,000 Baht had the highest MPH adherence (18.64), but this decreased as income increased. Spirits and MEAH (PC2) and PMTH (PC3) also decline as income levels rise.

FCP 2011, 2015 and 2019: Disaggregated data showed that adherence to PF and Spirits, TTM, and MVM decline as the household wealth increased, while TTM adherence remained within a certain range. Aggregated data on FCPs indicate that adherence to TTM and SFPF decreased as household income increases, while adherence to mixed patterns increased with wealth. VSF adherence remained within a certain range without a specific trend.

FCoP 2011, 2015 and 2019: Households with no income have the highest MPF adherence (61%), while other groups show variations between 55% and 57.5%. MVM adherence ranges between 70.8% and 71.8%, without a clear trend. TTMHS pattern adherence increased with wealth, although households with no income also have higher adherence (77.8%) compared to low–earning groups. According to disaggregated data, MSM adherence decreased as the income increases. Aggregated data for MEAH-PF patterns showed higher adherence for households with no income compared to low–income groups, with adherence increasing toward higher–income groups. TCPF and MEAH pattern adherence decreased as household wealth increased.

1.1.10. Household Food Consumption Expenses

FCP 2000–2021: The mixed pattern (PC1) showed a dramatic increase with higher food consumption expenses, peaking at 30.2 for expenses ≥50,001 Baht, indicating a strong correlation between food expenditure and primary household characteristics. Adherence to MPH (PC2) decreased as expenditures increase, while Spirits (PC3) adherence increased with higher food expenses.

FCoP 2000–2021: Households with higher food consumption expenses (>50,001 Baht) exhibit lower adherence to MPH (PC1) but significantly higher values for Spirits, MEAH (62.43) and PMTH (77.95). Households with the lowest food consumption expenses (1–5,000 Baht) have higher MPH adherence (17.82).

FCP 2011, 2015 and 2019: Disaggregated data showed that adherence to PF and Spirits decreased as household food expenses increase (from 27.4% to 16.5%), while households with zero food expenses have the second highest adherence rate (25.8%). The TTMM pattern has the lowest adherence among households spending more than 50,001 on food, whereas the MVM pattern showed the highest adherence (49.3%). The TTM pattern did not display a clear trend with values varying within a certain range. Aggregated data reveal that adherence to mixed patterns among households increased (from 0% to 40%) but showed the lowest value (16.4%) for households spending more than 50,001. Adherence to VSF and SFPF patterns increased with higher food expenses, while TTM adherence decreased as food expenditures rises (Table 2).

FCoP 2011, 2015 and 2019: Disaggregated data indicate that household adherence to TTMHS increased as food expenses rise. The MPF pattern showed a similar trend, except for the lowest adherence rate (54.9%) among households with expenses exceeding 50,001. The MSM pattern had the highest adherence for households with high food expenses, but no clear trend was observed for the MVM pattern. Aggregated data reveal that households with both zero and the highest food expenses (20,001–>50,001) showed the highest adherence to MEAH-PF. The MPF, MVM, and MSM patterns do not follow a consistent trend with adherence varying within a certain range (Table 2).

Table 1. Mean adherence to household food consumption (FCPs) and composition patterns (FCoPs) generated using (2000–2021), food consumption data by selected household characteristics.

| **HH characteristics** | | **No of Houses** | **Food Consumption 2000**–**2021** | | | **Food Composition 2000**–**2021** | | |
| --- | --- | --- | --- | --- | --- | --- | --- | --- |
|  |  |  | **Mixed pattern** | **Meals Prepared at Home** | **Spirits** | **Meals Prepared at Home** | **Sprits And Meals Away Home** | **Prepared Meals Taken Home** |
| Year | 2000–2021 | 804,110 | 2.13 (0) | 54.65 (0) | 21.35 (0) | 15.94 (0.01) | 50.12 (0.01) | 78.5 (0.01) |
|  | 2000 | 24,747 | 4.83 (0.02) | 54.73 (0.01) | 21.43 (0.01) | 18.35 (0.04) | 51.1 (0.04) | 75.9 (0.04) |
|  | 2006 | 44,918 | 1.32 (0) | 54.66 (0) | 21.4 (0) | 15.65 (0.03) | 50.08 (0.03) | 76.29 (0.03) |
|  | 2010 | 44,273 | 1.55 (0) | 54.64 (0) | 21.37 (0) | 15.26 (0.03) | 50.34 (0.03) | 77.84 (0.03) |
|  | 2015 | 43,400 | 1.98 (0.01) | 54.76 (0) | 21.33 (0) | 16.69 (0.03) | 49.76 (0.03) | 81.17 (0.03) |
|  | 2021 | 46,840 | 2.03 (0.01) | 54.62 (0) | 21.33 (0) | 15.93 (0.03) | 48.9 (0.03) | 78.56 (0.03) |
| Region | 1–BMR | 46,902 | 2.38 (0.01) | 54.11 (0) | 21.27 (0) | 9.68 (0.03) | 50.1 (0.03) | 78.92 (0.03) |
|  | 2–Central (Exclude BMR) | 232,609 | 2.22 (0) | 54.53 (0) | 21.35 (0) | 14.42 (0.01) | 49.47 (0.01) | 77.17 (0.01) |
|  | 3–North | 192,794 | 1.86 (0) | 54.77 (0) | 21.38 (0) | 17.97 (0.02) | 50.65 (0.01) | 78.92 (0.02) |
|  | 4–Northeast | 211,020 | 2.06 (0) | 54.79 (0) | 21.37 (0) | 17.02 (0.01) | 51.23 (0.01) | 80.33 (0.01) |
|  | 5–South | 120,785 | 2.41 (0.01) | 54.65 (0) | 21.32 (0) | 16.15 (0.02) | 48.6 (0.02) | 77.01 (0.02) |
| Community | Urban | 482,426 | 2.1 (0) | 54.54 (0) | 21.34 (0) | 14.38 (0.01) | 49.83 (0.01) | 78.65 (0.01) |
|  | Rural | 321,684 | 2.16 (0) | 54.81 (0) | 21.38 (0) | 18.28 (0.01) | 50.57 (0.01) | 78.27 (0.01) |
| HH Head Gender | Male | 483,350 | 2.08 (0) | 54.65 (0) | 21.37 (0) | 15.92 (0.01) | 50.71 (0.01) | 78.58 (0.01) |
|  | Female | 285,975 | 1.84 (0) | 54.64 (0) | 21.33 (0) | 15.69 (0.01) | 49.07 (0.01) | 78.5 (0.01) |
|  | #N/A | 34,785 | 5.1 (0.62) | 54.7 (0.03) | 21.3 (0.02) | 18.24 (0.12) | 50.67 (0.02) | 77.32 (0.03) |
| HH Head Age | 9–25 | 66,882 | 1.46 (0) | 54.54 (0) | 21.36 (0) | 13.03 (0.03) | 50.59 (0.03) | 77.41 (0.03) |
|  | 26–55 | 384,989 | 2.11 (0) | 54.59 (0) | 21.36 (0) | 14.96 (0.01) | 50.57 (0.01) | 77.94 (0.01) |
|  | 56–65 | 160,882 | 2.06 (0) | 54.72 (0) | 21.35 (0) | 17.31 (0.02) | 49.92 (0.01) | 79.18 (0.02) |
|  | 66–100 | 156,572 | 1.86 (0) | 54.75 (0) | 21.34 (0) | 17.66 (0.02) | 48.91 (0.02) | 79.9 (0.02) |
|  | #N/A | 34,785 | 5.12 (0.02) | 54.74 (0.01) | 21.33 (0) | 18.24 (0.04) | 50.68 (0.03) | 77.32 (0.03) |
| HH Head Education | Informal education | 4,550 | 4.34 (0.04) | 54.97 (0.01) | 21.32 (0.01) | 20.76 (0.1) | 49.73 (0.09) | 77.39 (0.11) |
|  | Pre and primary education | 461,495 | 2 (0) | 54.74 (0) | 21.36 (0) | 17.12 (0.01) | 50.21 (0.01) | 78.74 (0.01) |
|  | Secondary (lower, upper and post) education | 109,562 | 2.31 (0.01) | 54.48 (0) | 21.35 (0) | 13.26 (0.02) | 50.24 (0.02) | 77.59 (0.02) |
|  | Vocational education | 7,977 | 4.97 (0.04) | 54.02 (0.01) | 21.39 (0.01) | 12.03 (0.08) | 51.04 (0.08) | 74.97 (0.07) |
|  | Bachelor Degree and Diploma degree upper Bachelor | 72,976 | 2.3 (0.01) | 54.3 (0) | 21.32 (0) | 11.12 (0.02) | 49.96 (0.03) | 77.05 (0.02) |
|  | Master degree | 10,129 | 2.42 (0.02) | 54.17 (0.01) | 21.32 (0.01) | 10.55 (0.06) | 50.31 (0.07) | 76.93 (0.06) |
|  | Doctoral degree | 529 | 2.81 (0.12) | 53.93 (0.04) | 21.21 (0.02) | 9.12 (0.25) | 50.05 (0.31) | 76.56 (0.28) |
|  | Occupational Education (Medicine and Teaching) | 1,171 | 5.73 (0.09) | 54.27 (0.04) | 21.4 (0.04) | 15.1 (0.2) | 50.31 (0.22) | 75.16 (0.18) |
|  | Other education | 2,043 | 1.71 (0.03) | 54.67 (0.01) | 21.34 (0.01) | 16.37 (0.17) | 50.31 (0.12) | 78.57 (0.14) |
|  | #N/A | 133,678 | 2.03 (0) | 54.75 (0) | 21.35 (0) | 17.18 (0.02) | 49.76 (0.02) | 79.59 (0.02) |
| No eco act Members | 0 | 90,212 | 1.35 (0) | 54.67 (0) | 21.32 (0) | 15.88 (0.03) | 47.3 (0.02) | 80.35 (0.03) |
|  | 1–2 | 581,233 | 2.11 (0) | 54.64 (0) | 21.36 (0) | 15.76 (0.01) | 50.3 (0.01) | 78.32 (0.01) |
|  | 3–5 | 130,625 | 2.71 (0.01) | 54.69 (0) | 21.36 (0) | 16.75 (0.02) | 51.28 (0.01) | 78.05 (0.02) |
|  | 6–8 | 2,011 | 4.2 (0.08) | 54.54 (0.02) | 21.32 (0.01) | 15.84 (0.13) | 51.57 (0.12) | 76.79 (0.14) |
|  | 9–14 | 29 | 6.59 (1.35) | 54.59 (0.27) | 21.32 (0.13) | 16.57 (1.22) | 52.82 (0.98) | 75.9 (1.12) |
| No Members | 1–3 | 527,795 | 1.68 (0) | 54.62 (0) | 21.37 (0) | 15.46 (0.01) | 49.77 (0.01) | 79.45 (0.01) |
|  | 4–6 | 252,372 | 2.87 (0) | 54.71 (0) | 21.34 (0) | 16.85 (0.01) | 50.78 (0.01) | 76.78 (0.01) |
|  | 7–10 | 23,159 | 4.06 (0.02) | 54.73 (0.01) | 21.3 (0) | 16.84 (0.03) | 51.02 (0.03) | 75.64 (0.04) |
|  | 11–15 | 784 | 6.22 (0.2) | 54.6 (0.05) | 21.22 (0.02) | 16.16 (0.18) | 51.21 (0.16) | 74.66 (0.22) |
| HH Income | 0 | 314,068 | 1.72 (0) | 54.64 (0) | 21.36 (0) | 15.36 (0.01) | 50.39 (0.01) | 78.49 (0.01) |
|  | 1–10,000 | 161,428 | 2.02 (0) | 54.85 (0) | 21.38 (0) | 18.64 (0.02) | 50.08 (0.02) | 79.25 (0.02) |
|  | 10,001–20,000 | 155,126 | 2.28 (0) | 54.69 (0) | 21.36 (0) | 16.19 (0.02) | 49.95 (0.02) | 78.46 (0.02) |
|  | 20,001–50,000 | 133,680 | 2.69 (0.01) | 54.52 (0) | 21.33 (0) | 14.54 (0.02) | 49.82 (0.02) | 77.92 (0.02) |
|  | 50,001–100,001 | 31,264 | 3.16 (0.01) | 54.31 (0.01) | 21.31 (0) | 13.27 (0.04) | 49.85 (0.04) | 77.53 (0.03) |
|  | 100,001–200,000 | 6,271 | 3.73 (0.04) | 54.11 (0.01) | 21.27 (0.01) | 12.58 (0.08) | 49.9 (0.08) | 77.47 (0.08) |
|  | 200,001–400,000 | 1,029 | 4.28 (0.15) | 53.91 (0.06) | 21.27 (0.04) | 12.65 (0.21) | 49.83 (0.21) | 77.51 (0.2) |
|  | >= 400,001 | 431 | 4.14 (0.24) | 53.78 (0.14) | 21.48 (0.19) | 12.75 (0.31) | 49.29 (0.34) | 77.6 (0.31) |
|  | **#N/A** | 813 | 2.06 (0.04) | 54.71 (0.02) | 21.34 (0.01) | 17.73 (0.25) | 49.41 (0.21) | 79.54 (0.23) |
| **HH Food Consumption Expenses** | 0 | 187 | 0.05 (0.03) | 54.74 (0.01) | 21.41 (0) | 8.64 (0.07) | 54.7 (0.08) | 80.72 (0.03) |
|  | 1–5000 | 377,120 | 1.62 (0) | 54.8 (0) | 21.37 (0) | 17.82 (0.01) | 49.71 (0.01) | 79.36 (0.01) |
|  | 5001–10,000 | 324,718 | 2.29 (0) | 54.63 (0) | 21.34 (0) | 14.95 (0.01) | 50.24 (0.01) | 77.99 (0.01) |
|  | 10,001–15,000 | 94,053 | 3.31 (0.01) | 54.27 (0) | 21.32 (0) | 12.39 (0.02) | 51.09 (0.02) | 76.96 (0.02) |
|  | 15,001–20,000 | 4,867 | 4.72 (0.04) | 53.53 (0.02) | 21.36 (0.01) | 9.69 (0.07) | 52.9 (0.1) | 76.51 (0.08) |
|  | 20,001–25,000 | 3,004 | 6.07 (0.07) | 52.78 (0.03) | 21.55 (0.04) | 8.54 (0.09) | 54.32 (0.14) | 75.97 (0.11) |
|  | 25,001–50,000 | 145 | 12.6 (1.04) | 51 (0.62) | 22.59 (0.58) | 8.75 (0.62) | 56.15 (1.11) | 74.82 (0.99) |
|  | >=50,001 | 10 | 30.19 (6.84) | 42.85 (6.79) | 32.95 (7.75) | 7.73 (2.68) | 62.43 (4.15) | 77.95 (2.44) |
|  | **#N/A** | 6 | 79.63 (7.57) | 63.6 (16.13) | 19.09 (4.04) | 22.67 (5.42) | 44.83 (8.95) | 88.03 (3.88) |

Table 2. Mean adherence to household food consumption (FCPs) and composition patterns (FCoPs) using disaggregated and aggregated detailed food data (2011, 2015, and 2019) by selected household characteristics.

| **HH characteristics** | | **No of Houses** | **Disaggregated 2011**–**2019** | | | | **Aggregated 2011**–**2019** | | | | **Disaggregated 2011**–**2019** | | | | **Aggregated 2011**–**2019** | | | |
| --- | --- | --- | --- | --- | --- | --- | --- | --- | --- | --- | --- | --- | --- | --- | --- | --- | --- | --- |
|  |  |  | **PF and Spirits PC1** | **TTMM PC2** | **TTM PC3** | **MVM PC4** | **MP PC1** | **TTM PC2** | **VSF PC3** | **SFPF PC4** | **TTMHS PC1** | **MPF PC2** | **MVM PC3** | **MSM PC4** | **MEAH**–**PF** | **TTM PC2** | **TCPF PC3** | **MEAH PC4** |
| Year | 2010 | 131,069 | 25.65 (0.03) | 56.99 (0.02) | 71.62 (0.01) | 47.03 (0.02) | 8.14 (0.02) | 57.96 (0.01) | 78.46 (0.01) | 57.66 (0.01) | 74.35 (0.03) | 56.99 (0.02) | 71.62 (0.01) | 47.04 (0.02) | 80.88 (0.03) | 25.14 (0.01) | 24.45 (0.01) | 57.84 (0.01) |
|  | 2011 | 42,083 | 26.93 (0.05) | 48.78 (0.03) | 73.39 (0.03) | 48.11 (0.03) | 7.28 (0.03) | 58.93 (0.02) | 77.65 (0.01) | 58.84 (0.02) | 73.07 (0.05) | 48.78 (0.03) | 73.39 (0.03) | 48.11 (0.03) | 79.53 (0.05) | 26.06 (0.03) | 22.49 (0.02) | 59.31 (0.02) |
|  | 2015 | 43,400 | 25.18 (0.05) | 60.65 (0.03) | 69.96 (0.02) | 47.24 (0.03) | 8.53 (0.04) | 57.53 (0.03) | 78.64 (0.01) | 57.2 (0.02) | 74.82 (0.05) | 60.65 (0.03) | 69.96 (0.02) | 47.24 (0.03) | 81.43 (0.05) | 24.9 (0.02) | 25.3 (0.02) | 57.22 (0.02) |
|  | 2019 | 45,586 | 24.95 (0.05) | 60.89 (0.03) | 71.62 (0.02) | 45.85 (0.03) | 8.55 (0.04) | 57.49 (0.03) | 79.01 (0.01) | 57.04 (0.02) | 75.05 (0.05) | 60.89 (0.03) | 71.62 (0.02) | 45.85 (0.03) | 81.57 (0.05) | 24.53 (0.02) | 25.4 (0.02) | 57.11 (0.02) |
| Region | 1–BMR | 7,422 | 19.92 (0.08) | 57.79 (0.08) | 71.45 (0.04) | 45.85 (0.05) | 8.42 (0.09) | 55.09 (0.06) | 79 (0.03) | 58.21 (0.04) | 80.08 (0.08) | 57.79 (0.08) | 71.45 (0.04) | 45.86 (0.05) | 86.45 (0.08) | 25.41 (0.04) | 24.44 (0.04) | 57.44 (0.05) |
|  | 2–Central (Exclude BMR) | 37,870 | 23.72 (0.05) | 57.12 (0.04) | 71.28 (0.02) | 45.63 (0.03) | 8.85 (0.04) | 57.11 (0.03) | 78.49 (0.01) | 58.12 (0.02) | 76.28 (0.05) | 57.12 (0.04) | 71.28 (0.02) | 45.63 (0.03) | 82.75 (0.05) | 25.57 (0.02) | 24.07 (0.02) | 57.43 (0.02) |
|  | 3–North | 31,468 | 28.94 (0.06) | 56.96 (0.06) | 71.89 (0.03) | 48.46 (0.03) | 7.36 (0.03) | 59.4 (0.02) | 78.33 (0.01) | 57.12 (0.02) | 71.06 (0.06) | 56.96 (0.06) | 71.89 (0.03) | 48.46 (0.03) | 77.84 (0.05) | 24.88 (0.02) | 24.65 (0.02) | 58.75 (0.02) |
|  | 4–Northeast | 34,537 | 28.45 (0.05) | 57.97 (0.06) | 72.31 (0.04) | 50.11 (0.03) | 7.31 (0.03) | 59.17 (0.02) | 78.56 (0.01) | 56.25 (0.02) | 71.55 (0.05) | 57.97 (0.06) | 72.31 (0.04) | 50.11 (0.03) | 78.29 (0.05) | 24.19 (0.03) | 25.71 (0.02) | 58.45 (0.02) |
|  | 5–South | 19,772 | 25.03 (0.06) | 53.78 (0.07) | 70.66 (0.03) | 42.65 (0.04) | 9.19 (0.05) | 58.08 (0.03) | 77.79 (0.02) | 59.93 (0.03) | 74.97 (0.06) | 53.78 (0.07) | 70.66 (0.03) | 42.65 (0.04) | 81 (0.06) | 26.33 (0.03) | 22.27 (0.03) | 56.56 (0.03) |
| Community | Urban | 78,155 | 22.41 (0.04) | 57.87 (0.03) | 71.59 (0.02) | 46.48 (0.02) | 8.18 (0.04) | 56.53 (0.02) | 78.74 (0.01) | 57.81 (0.02) | 77.59 (0.04) | 57.87 (0.03) | 71.59 (0.02) | 46.48 (0.02) | 84.04 (0.04) | 25.12 (0.02) | 24.51 (0.02) | 57.64 (0.02) |
|  | Rural | 52,914 | 28.01 (0.04) | 56.34 (0.04) | 71.64 (0.02) | 47.44 (0.02) | 8.11 (0.03) | 59 (0.01) | 78.25 (0.01) | 57.55 (0.01) | 71.99 (0.04) | 56.34 (0.04) | 71.64 (0.02) | 47.44 (0.02) | 78.58 (0.04) | 25.15 (0.02) | 24.4 (0.02) | 57.99 (0.02) |
| HH Head Gender | Male | 80,644 | 25.61 (0.04) | 56.83 (0.03) | 71.73 (0.02) | 47.19 (0.02) | 8.35 (0.03) | 57.99 (0.02) | 78.45 (0.01) | 57.65 (0.01) | 74.39 (0.04) | 56.83 (0.03) | 71.73 (0.02) | 47.19 (0.02) | 80.92 (0.04) | 25.08 (0.02) | 24.42 (0.01) | 58.09 (0.02) |
|  | Female | 50,425 | 25.71 (0.05) | 57.26 (0.04) | 71.42 (0.02) | 46.77 (0.03) | 7.78 (0.03) | 57.89 (0.02) | 78.47 (0.01) | 57.69 (0.02) | 74.29 (0.05) | 57.26 (0.04) | 71.42 (0.02) | 46.77 (0.03) | 80.82 (0.05) | 25.24 (0.02) | 24.5 (0.02) | 57.41 (0.02) |
| HH Head Age | 9–25 | 3,837 | 17.37 (0.14) | 57.52 (0.11) | 71.21 (0.04) | 47.81 (0.06) | 4.15 (0.08) | 55.88 (0.06) | 78.49 (0.02) | 57.35 (0.04) | 82.63 (0.14) | 57.52 (0.11) | 71.21 (0.04) | 47.81 (0.06) | 88.83 (0.14) | 25.56 (0.04) | 25.19 (0.06) | 59.18 (0.1) |
|  | 26–55 | 66,666 | 24.27 (0.04) | 56.68 (0.03) | 71.63 (0.02) | 47.01 (0.02) | 8.3 (0.03) | 57.47 (0.02) | 78.39 (0.01) | 57.65 (0.01) | 75.73 (0.04) | 56.68 (0.03) | 71.63 (0.02) | 47.01 (0.02) | 82.18 (0.04) | 25.24 (0.02) | 24.39 (0.01) | 58 (0.02) |
|  | 56–65 | 30,722 | 27.51 (0.06) | 57.44 (0.06) | 71.71 (0.03) | 47.05 (0.04) | 8.72 (0.04) | 58.58 (0.03) | 78.53 (0.02) | 57.61 (0.02) | 72.49 (0.06) | 57.44 (0.06) | 71.71 (0.03) | 47.05 (0.04) | 79.09 (0.06) | 24.89 (0.03) | 24.51 (0.02) | 57.62 (0.03) |
|  | 66–100 | 29,844 | 28.29 (0.06) | 57.18 (0.06) | 71.54 (0.04) | 46.96 (0.04) | 7.73 (0.05) | 58.8 (0.03) | 78.53 (0.02) | 57.79 (0.02) | 71.71 (0.06) | 57.18 (0.06) | 71.54 (0.04) | 46.96 (0.04) | 78.41 (0.06) | 25.07 (0.04) | 24.43 (0.03) | 57.48 (0.03) |
| HH Head Education | Informal education | 59,357 | 25.52 (0.05) | 60.05 (0.03) | 70.17 (0.02) | 47.21 (0.03) | 8.35 (0.04) | 57.69 (0.02) | 78.61 (0.01) | 57.27 (0.02) | 74.48 (0.05) | 60.05 (0.03) | 70.17 (0.02) | 47.21 (0.03) | 81.1 (0.05) | 24.95 (0.02) | 25.17 (0.02) | 57.33 (0.02) |
|  | Pre and primary education | 12,872 | 27.28 (0.04) | 54.8 (0.04) | 72.6 (0.02) | 47.23 (0.03) | 7.84 (0.03) | 58.86 (0.02) | 78.28 (0.01) | 57.77 (0.02) | 72.72 (0.04) | 54.8 (0.04) | 72.6 (0.02) | 47.24 (0.03) | 79.27 (0.04) | 25.2 (0.02) | 24.04 (0.02) | 58.26 (0.02) |
|  | Secondary (lower, upper and post) education | 144 | 22.12 (0.09) | 56.1 (0.08) | 72.08 (0.04) | 46.36 (0.05) | 8.3 (0.07) | 56.55 (0.05) | 78.48 (0.03) | 57.96 (0.04) | 77.88 (0.09) | 56.1 (0.08) | 72.08 (0.04) | 46.36 (0.05) | 84.18 (0.09) | 25.41 (0.04) | 24.12 (0.04) | 57.84 (0.05) |
|  | Vocational education | 8,866 | 15.24 (0.48) | 56.88 (0.59) | 71.42 (0.2) | 48.41 (0.32) | 3.57 (0.53) | 55.25 (0.33) | 78.37 (0.13) | 57.83 (0.64) | 84.76 (0.48) | 56.88 (0.59) | 71.42 (0.2) | 48.42 (0.32) | 90.74 (0.45) | 25.75 (0.13) | 25.1 (0.39) | 60.26 (0.44) |
|  | Bachelor Degree and Diploma degree upper Bachelor | 1,293 | 20.19 (0.1) | 56.39 (0.1) | 72.18 (0.04) | 45.75 (0.06) | 8.68 (0.1) | 55.28 (0.07) | 78.74 (0.03) | 58.6 (0.05) | 79.81 (0.1) | 56.39 (0.1) | 72.18 (0.04) | 45.75 (0.06) | 86.05 (0.1) | 25.39 (0.04) | 23.79 (0.04) | 57.77 (0.06) |
|  | Master degree | 66 | 20.25 (0.24) | 55.77 (0.23) | 72.2 (0.11) | 45.49 (0.17) | 10.35 (0.28) | 54.5 (0.21) | 78.78 (0.11) | 59.17 (0.15) | 79.75 (0.24) | 55.77 (0.23) | 72.2 (0.11) | 45.49 (0.17) | 86 (0.23) | 25.53 (0.11) | 23.43 (0.1) | 57.73 (0.16) |
|  | Doctoral degree | 336 | 18.29 (0.78) | 57.98 (0.74) | 71.96 (0.3) | 44.32 (0.59) | 12.02 (1.37) | 52.1 (0.66) | 79.47 (0.37) | 61.03 (0.81) | 81.71 (0.78) | 57.98 (0.74) | 71.96 (0.3) | 44.32 (0.59) | 87.85 (0.8) | 25.2 (0.23) | 23.39 (0.37) | 57.7 (0.83) |
|  | Occupational Education (Medicine and Teaching) | 48,135 | 24.7 (0.74) | 56.52 (0.54) | 72.21 (0.25) | 43.86 (0.35) | 7.96 (0.55) | 58.7 (0.24) | 78.74 (0.15) | 58.68 (0.2) | 75.3 (0.74) | 56.52 (0.54) | 72.21 (0.25) | 43.86 (0.35) | 81.68 (0.69) | 25.1 (0.22) | 23.44 (0.22) | 57.84 (0.26) |
|  | Other education | 17,221 | 26.85 (0.09) | 57.45 (0.08) | 71.26 (0.05) | 47.03 (0.06) | 5.34 (0.05) | 58.31 (0.03) | 78.57 (0.01) | 57.79 (0.02) | 73.15 (0.09) | 57.45 (0.08) | 71.26 (0.05) | 47.03 (0.06) | 79.91 (0.09) | 25.31 (0.05) | 24.77 (0.04) | 57.23 (0.04) |
|  | #N/A | 92,525 |  |  |  |  |  |  |  |  |  |  |  |  |  |  |  |  |
| No eco act Members | 0 | 17,221 | 26.85 (0.09) | 57.45 (0.08) | 71.26 (0.05) | 47.03 (0.06) | 5.34 (0.05) | 58.31 (0.03) | 78.57 (0.01) | 57.79 (0.02) | 73.15 (0.09) | 57.45 (0.08) | 71.26 (0.05) | 47.03 (0.06) | 79.91 (0.09) | 25.31 (0.05) | 24.77 (0.04) | 57.23 (0.04) |
|  | 1–2 | 92,525 | 25.15 (0.04) | 57.06 (0.03) | 71.59 (0.02) | 47.05 (0.02) | 7.86 (0.02) | 57.77 (0.02) | 78.45 (0.01) | 57.64 (0.01) | 74.85 (0.04) | 57.06 (0.03) | 71.59 (0.02) | 47.05 (0.02) | 81.34 (0.04) | 25.16 (0.01) | 24.48 (0.01) | 57.93 (0.02) |
|  | 3–5 | 21,063 | 26.91 (0.06) | 56.37 (0.07) | 71.99 (0.04) | 46.98 (0.04) | 11.26 (0.06) | 58.47 (0.04) | 78.42 (0.02) | 57.67 (0.03) | 73.09 (0.06) | 56.37 (0.07) | 71.99 (0.04) | 46.98 (0.04) | 79.64 (0.06) | 24.91 (0.03) | 24.08 (0.03) | 57.92 (0.03) |
|  | 6–8 | 258 | 26.61 (0.54) | 54.75 (0.66) | 72.49 (0.32) | 46.97 (0.39) | 15.21 (0.72) | 58.82 (0.34) | 78.21 (0.23) | 57.73 (0.36) | 73.39 (0.54) | 54.75 (0.66) | 72.49 (0.32) | 46.97 (0.39) | 79.93 (0.54) | 25.03 (0.24) | 23.67 (0.23) | 58.43 (0.22) |
|  | 9–14 | 2 | 25.42 (1.48) | 47.84 (0.34) | 68.83 (3.22) | 48.23 (0.35) | 11.56 (1.03) | 58.33 (0.55) | 75.42 (0.51) | 58.14 (0.5) | 74.58 (1.48) | 47.84 (0.34) | 68.83 (3.22) | 48.24 (0.35) | 80.44 (2.14) | 29.75 (2.42) | 24.46 (0.94) | 58.98 (1.73) |
| No Members | 1–3 | 91,117 | 25.24 (0.04) | 57.33 (0.03) | 71.49 (0.02) | 47.15 (0.02) | 6.69 (0.02) | 57.83 (0.02) | 78.51 (0.01) | 57.62 (0.01) | 74.76 (0.04) | 57.33 (0.03) | 71.49 (0.02) | 47.15 (0.02) | 81.3 (0.04) | 25.18 (0.02) | 24.64 (0.01) | 57.9 (0.02) |
|  | 4–6 | 36,906 | 26.54 (0.04) | 56.29 (0.05) | 71.89 (0.03) | 46.8 (0.03) | 11.06 (0.04) | 58.21 (0.03) | 78.37 (0.02) | 57.74 (0.02) | 73.46 (0.04) | 56.29 (0.05) | 71.89 (0.03) | 46.8 (0.03) | 79.95 (0.04) | 25.03 (0.02) | 24.04 (0.02) | 57.71 (0.02) |
|  | 7–10 | 2,969 | 26.62 (0.14) | 55.52 (0.19) | 71.83 (0.1) | 46.66 (0.1) | 14.46 (0.18) | 58.57 (0.11) | 78.03 (0.07) | 57.75 (0.11) | 73.38 (0.14) | 55.52 (0.19) | 71.83 (0.1) | 46.66 (0.1) | 79.87 (0.13) | 25.25 (0.09) | 23.81 (0.07) | 57.78 (0.07) |
|  | 11–15 | 77 | 26.36 (0.79) | 55.68 (0.91) | 71.1 (0.57) | 45.86 (0.62) | 18.27 (1.46) | 59.23 (0.59) | 77.92 (0.51) | 58.48 (0.81) | 73.64 (0.79) | 55.68 (0.91) | 71.1 (0.57) | 45.87 (0.62) | 80.12 (0.76) | 25.75 (0.44) | 23.47 (0.44) | 57.67 (0.35) |
| HH Income | 0 | 4 | 22.18 (0.87) | 60.96 (2.84) | 70.8 (0.27) | 45.64 (0.63) | 10.1 (0.18) | 55.41 (0.77) | 78.66 (0.29) | 56.54 (0.83) | 77.82 (0.87) | 60.96 (2.84) | 70.8 (0.27) | 45.64 (0.63) | 84.14 (0.56) | 24.63 (0.46) | 25.18 (0.93) | 53.65 (0.81) |
|  | 1–10,000 | 32,281 | 28.92 (0.06) | 55.9 (0.06) | 71.55 (0.04) | 48.53 (0.04) | 5.08 (0.03) | 59.26 (0.02) | 78.28 (0.01) | 57.44 (0.01) | 71.08 (0.06) | 55.9 (0.06) | 71.55 (0.04) | 48.53 (0.04) | 77.8 (0.06) | 25.31 (0.04) | 24.86 (0.03) | 58.46 (0.03) |
|  | 10,001–20,000 | 4,455 | 26.09 (0.05) | 57.12 (0.05) | 71.65 (0.02) | 47.33 (0.03) | 7.28 (0.03) | 58.63 (0.02) | 78.33 (0.01) | 57.38 (0.02) | 73.91 (0.05) | 57.12 (0.05) | 71.65 (0.02) | 47.33 (0.03) | 80.45 (0.05) | 25.05 (0.02) | 24.61 (0.02) | 57.96 (0.02) |
|  | 20,001–50,000 | 41,505 | 23.89 (0.05) | 57.55 (0.04) | 71.6 (0.02) | 46.23 (0.03) | 9.74 (0.04) | 57.27 (0.03) | 78.54 (0.01) | 57.71 (0.02) | 76.11 (0.05) | 57.55 (0.04) | 71.6 (0.02) | 46.23 (0.03) | 82.55 (0.05) | 25.08 (0.02) | 24.24 (0.02) | 57.45 (0.02) |
|  | 50,001–100,001 | 9,966 | 22.33 (0.09) | 57.46 (0.08) | 71.73 (0.04) | 45.18 (0.06) | 12.65 (0.1) | 55.26 (0.07) | 78.99 (0.04) | 58.76 (0.06) | 77.67 (0.09) | 57.46 (0.08) | 71.73 (0.04) | 45.18 (0.06) | 84.01 (0.08) | 25.11 (0.04) | 23.7 (0.04) | 57.2 (0.05) |
|  | 100,001–200,000 | 2,041 | 21.17 (0.18) | 56.99 (0.19) | 71.71 (0.08) | 44.97 (0.12) | 14.55 (0.27) | 53.09 (0.21) | 79.28 (0.1) | 59.48 (0.14) | 78.83 (0.18) | 56.99 (0.19) | 71.71 (0.08) | 44.98 (0.12) | 85.05 (0.18) | 25.28 (0.08) | 23.58 (0.08) | 57.45 (0.12) |
|  | 200,001–400,000 | 337 | 21.8 (0.42) | 55.21 (0.51) | 71.45 (0.26) | 45.44 (0.29) | 13.81 (0.78) | 53.51 (0.62) | 78.58 (0.26) | 60.01 (0.41) | 78.2 (0.42) | 55.21 (0.51) | 71.45 (0.26) | 45.44 (0.29) | 84.5 (0.42) | 26.01 (0.29) | 22.99 (0.19) | 58.04 (0.29) |
|  | >= 400,001 | 141 | 21.88 (0.58) | 56.12 (0.72) | 71.18 (0.31) | 44.27 (0.49) | 15.56 (1.33) | 54.11 (0.73) | 78.64 (0.47) | 61.09 (0.69) | 78.12 (0.58) | 56.12 (0.72) | 71.18 (0.31) | 44.27 (0.49) | 84.36 (0.57) | 25.74 (0.35) | 22.73 (0.23) | 56.2 (0.46) |
|  | **#N/A** | 339 |  |  |  |  |  |  |  |  |  |  |  |  |  |  |  |  |
| HH Food Consumption Expenses | 0 | 129 | 25.8 (0) | 56.94 (0) | 71.59 (0) | 46.96 (0) | 0 (0) | 58.53 (0) | 78.6 (0) | 58.07 (0) | 74.2 (0) | 56.94 (0) | 71.59 (0) | 46.96 (0) | 87.09 (0) | 24.78 (0) | 24.06 (0) | 59.63 (0) |
|  | 1–5000 | 50,871 | 27.36 (0.06) | 55.9 (0.05) | 71.26 (0.03) | 48.02 (0.03) | 4.57 (0.02) | 58.81 (0.01) | 78.25 (0.01) | 57.6 (0.01) | 72.64 (0.06) | 55.9 (0.05) | 71.26 (0.03) | 48.02 (0.03) | 79.26 (0.06) | 25.67 (0.03) | 24.76 (0.02) | 58.35 (0.02) |
|  | 5001–10,000 | 61,364 | 25.34 (0.04) | 57.4 (0.04) | 71.84 (0.02) | 46.74 (0.02) | 8.86 (0.02) | 58.23 (0.02) | 78.4 (0.01) | 57.55 (0.02) | 74.66 (0.04) | 57.4 (0.04) | 71.84 (0.02) | 46.74 (0.02) | 81.16 (0.04) | 24.86 (0.01) | 24.33 (0.02) | 57.62 (0.02) |
|  | 10,001–15,000 | 17,622 | 22.51 (0.06) | 58.46 (0.06) | 71.84 (0.02) | 45.51 (0.04) | 14.37 (0.06) | 55.44 (0.05) | 79.06 (0.03) | 58.04 (0.05) | 77.49 (0.06) | 58.46 (0.06) | 71.83 (0.02) | 45.51 (0.04) | 83.86 (0.05) | 24.67 (0.02) | 24.03 (0.03) | 57.15 (0.03) |
|  | 15,001–20,000 | 718 | 20.04 (0.23) | 58.55 (0.19) | 71.83 (0.09) | 44.88 (0.19) | 21.28 (0.44) | 50.55 (0.35) | 79.94 (0.2) | 59.23 (0.33) | 79.96 (0.23) | 58.55 (0.19) | 71.83 (0.09) | 44.88 (0.19) | 86.13 (0.22) | 24.74 (0.08) | 23.84 (0.11) | 57.96 (0.18) |
|  | 20,001–25,000 | 347 | 19 (0.32) | 58.47 (0.26) | 72.14 (0.08) | 45.24 (0.22) | 25.77 (0.77) | 46.1 (0.57) | 81.29 (0.31) | 60.67 (0.46) | 81 (0.32) | 58.47 (0.26) | 72.14 (0.08) | 45.24 (0.22) | 87.16 (0.3) | 24.51 (0.1) | 23.71 (0.11) | 58.81 (0.28) |
|  | 25,001–50,000 | 17 | 17.11 (1.29) | 57.59 (0.65) | 72.08 (0.27) | 46.41 (0.94) | 40.13 (6.08) | 26.99 (3.67) | 82.72 (2.11) | 59.94 (8.81) | 82.89 (1.29) | 57.59 (0.65) | 72.08 (0.27) | 46.41 (0.94) | 89.69 (1.31) | 25.19 (0.2) | 24.15 (0.82) | 61.62 (1.47) |
|  | >=50,001 | 1 | 16.54 (0) | 54.9 (0) | 71.87 (0) | 49.29 (0) | 16.38 (0) | 35.65 (0) | 73.21 (0) | 59.46 (0) | 83.46 (0) | 54.9 (0) | 71.87 (0) | 49.29 (0) | 88.86 (0) | 26.49 (0) | 23.14 (0) | 64.14 (0) |
